# Breaking the computational barriers of pairwise genome comparison

**DOI:** 10.1186/s12859-015-0679-9

**Published:** 2015-08-11

**Authors:** Oscar Torreno, Oswaldo Trelles

**Affiliations:** Advanced Computing Technologies Unit, RISC Software GmbH, Softwarepark 35, Hagenberg, 4232 Austria; Computer Architecture Department, University of Malaga, Bulevar Luis Pasteur 35, Malaga, 29071 Spain

**Keywords:** Comparative genomics, Out-of-core algorithm, External memory, Long sequences comparison

## Abstract

**Background:**

Conventional pairwise sequence comparison software algorithms are being used to process much larger datasets than they were originally designed for. This can result in processing bottlenecks that limit software capabilities or prevent full use of the available hardware resources. Overcoming the barriers that limit the efficient computational analysis of large biological sequence datasets by retrofitting existing algorithms or by creating new applications represents a major challenge for the bioinformatics community.

**Results:**

We have developed C libraries for pairwise sequence comparison within diverse architectures, ranging from commodity systems to high performance and cloud computing environments. Exhaustive tests were performed using different datasets of closely- and distantly-related sequences that span from small viral genomes to large mammalian chromosomes. The tests demonstrated that our solution is capable of generating high quality results with a linear-time response and controlled memory consumption, being comparable or faster than the current state-of-the-art methods.

**Conclusions:**

We have addressed the problem of pairwise and all-versus-all comparison of large sequences in general, greatly increasing the limits on input data size. The approach described here is based on a modular out-of-core strategy that uses secondary storage to avoid reaching memory limits during the identification of High-scoring Segment Pairs (HSPs) between the sequences under comparison. Software engineering concepts were applied to avoid intermediate result re-calculation, to minimise the performance impact of input/output (I/O) operations and to modularise the process, thus enhancing application flexibility and extendibility. Our computationally-efficient approach allows tasks such as the massive comparison of complete genomes, evolutionary event detection, the identification of conserved synteny blocks and inter-genome distance calculations to be performed more effectively.

**Electronic supplementary material:**

The online version of this article (doi:10.1186/s12859-015-0679-9) contains supplementary material, which is available to authorized users.

## Background

The number of genome sequencing projects has grown exponentially, in parallel with a drastic reduction in the cost of sequencing. For example, at the turn of the millennium the cost of sequencing 1 Mbp of genomic DNA (million DNA base pairs) was about 10 thousand US dollars, compared to around 5 US cents at the time of writing [1]. Scientists are continuing to develop faster and cheaper methods that will allow the routine sequencing of individual patient genomes, thus truly ushering in the era of genetics-based personalised medicine.

The human genome is not the only one of interest to the research community, and the progression of sequencing technology also has huge consequences for studies involving the genomes of other organisms. At present, hundreds of different organisms, from all living kingdoms, have been sequenced and thousands more projects are on-going. These developments have put Comparative Genomics into the spotlight in order to provide the tools for studying relationships within this flood of data.

Pairwise sequence comparison algorithms have been implemented since the early days of bioinformatics. Original algorithms for global [2] and local alignments [3] were designed using dynamic programming techniques that result in quadratic calculation time and memory consumption proportional to the product of the total number of bases analysed.

When sequence analysis jumped from individual genes and proteins to full genomes, new software appeared, such as MegaBlast [4], MUMmer [5] and Gepard [6], the latter of which has been reported to be able to compare more than 300 Mbp of human chromosome-1 in approximately 1 h [6]. These software adopted some ideas introduced by the heuristic sequence database searching algorithms FASTA [7], and later BLAST [8]. These algorithms introduced a computational space reduction strategy based on the fast identification of matching points (hits) that are in turn used as seed points for the extension of local alignments. In FASTA, these matching points are perfect matches between K-mers (words of length k) from each sequence, while BLAST allows certain mismatches, thus enhancing its sensitivity. Other computational space reduction strategies confine the search to the most probable matching space (FASTA), or limit seed extension to regions with a minimal concentration of hits (BLAST).

Additionally, some of the previous software adopted other ideas coming from the string matching field such as the Generalized Suffix Trees and Suffix Array data structures [9, 10] which reduce significantly the computational complexity but still involves the use of significant memory resources (see Section 2.2 of the Additional file 1). In order to overcome the mentioned memory issue, a number of disk-based implementations were developed [11–13]. Despite using customised strategies to minimize the I/O operation overhead, they reported higher execution times for indexing the Human genome (6 h in [12] and 11 h in [13]) compared to 3 h for our proposed indexing strategy.

In general, the reference software was designed to deal with genes, proteins and small genome sequences and since are now used for much larger datasets than they were originally designed for, they are now reaching their limit in terms of memory capacity and efficient computation on single-CPU systems. Consequently, there is a pressing need to design new software that tackles the memory consumption problem caused by the analysis of very large genome sequence datasets. A good strategy to deal with this problem is to move data that does not fit into internal memory to external memory (i.e. hard disks), following what is known as an out-of-core strategy [14, 15]. However, since there is a difference of several orders of magnitude in access time between the two memory layers, special care must be taken in order to avoid performance degradation. Some of these approaches have previously been applied to bioinformatics [16], but not specifically for pairwise genome comparison.

In this document we report on GECKO (GEnome Comparison with K-mers Out-of-core), a modular application designed to identify collections of HSPs by pairwise genome comparison procedures, that can then be used to obtain gapped fragments. Our work improves on previous methods by introducing controlled memory usage and a modular design that allows further comparisons to be performed without the need to recalculate intermediate results and thus without sacrificing performance. We have benchmarked the application in terms of both performance and results quality. We designed experiments with datasets ranging from short sequences in the kilobase range to larger sequences up to 200 Mbp in length in order to compare GECKO against the best currently available software under both unfavourable and favourable conditions respectively. In addition, we performed a massive comparison exercise between mammalian chromosome sequences in order to test one of the key improvements of the application: the avoidance of intermediate result re-calculation. In the tests with short sequences, GECKO was slower compared to existing software, but with long sequences, the results were comparable or superior in terms of performance. The results quality in both cases (short and long sequences) was superior. Binaries are available from http://bitlab-es.com/gecko/. Source code is available from: https://sourceforge.net/projects/gecko-aligner/.

## Methods

**Fig. 1:**
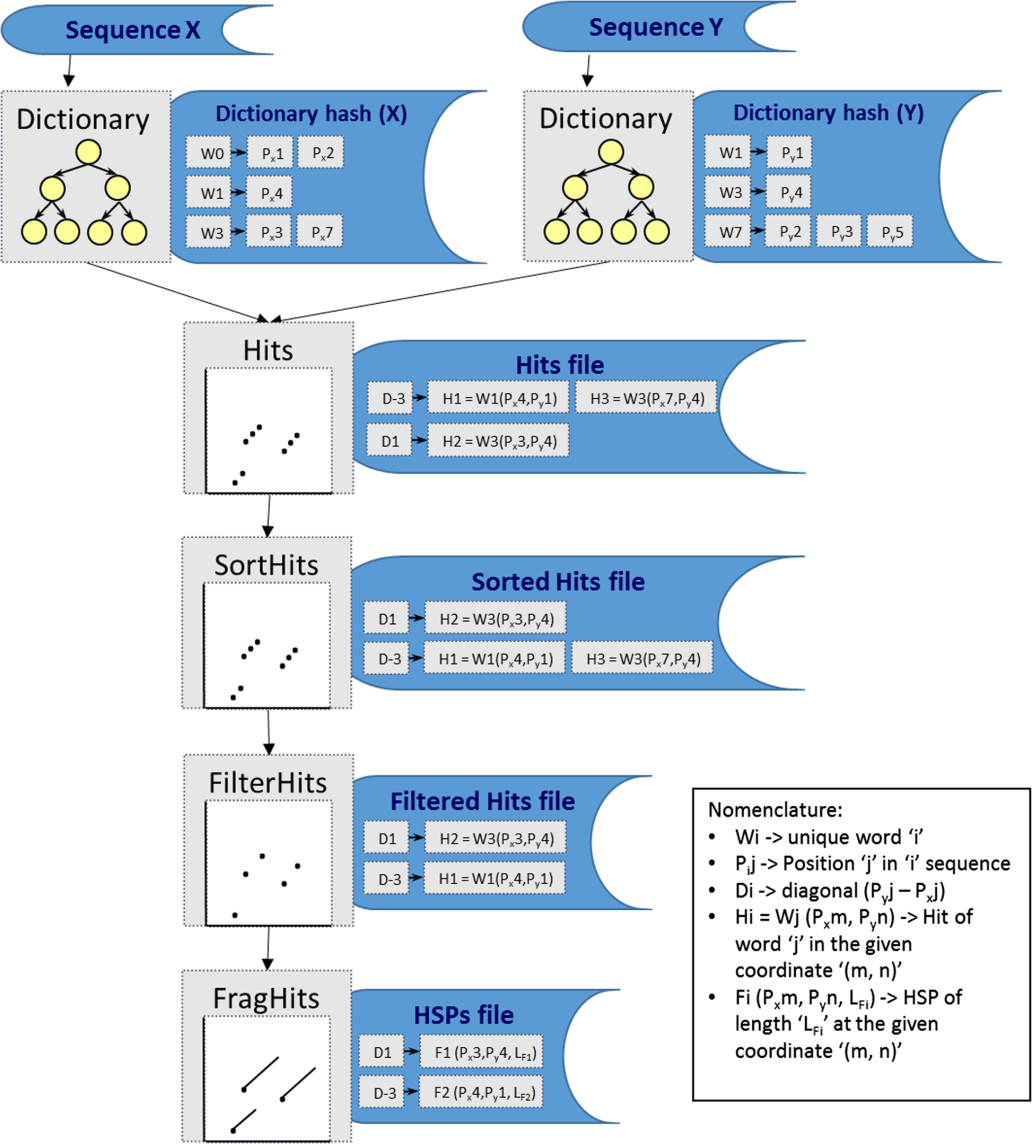
Summary of GECKO’s modular design. The branches on the top represent dictionary computation using the binary tree for each sequence. Once the dictionaries are calculated, perfect matches between words produce a set of seed points (hits). Afterwards, hits are sorted (by diagonal and offset inside the diagonal) and filtered. Finally, the hits are extended to generate a set of HSPs (FragHits). An additional figure with a real example is provided in Section 2.1 of the Additional file 1

To overcome the limitations of existing sequence comparison methods we focused firstly on the application-specific reduction of main memory and computational space usage, and secondly on modularising the process using classical software engineering concepts. In the next sections, we explain how we reduce memory usage using an out-of-core strategy designed to manage data structures that are too large to fit into main memory at one time. Naturally, memory management could be delegated to the Operating System using virtual memory concepts; however poorer data locality can result in performance degradation in memory intensive applications such as large-scale sequence analyses. In addition, we explain the strategies applied to the design of GECKO (see Fig. 1): (a) Dictionary calculation, (b) Hits determination, (c) HSP detection, and (d) HSP post-processing.

### Memory consumption and computational space reduction

This section describes our approaches for dealing with the memory usage problem with an out-of-core solution, while compensating for the slower access time of secondary storage devices in several ways:

1. Sensitivity studies involve obtaining results for different *K* values (word sizes) and require computing word dictionaries for each value. It is easy to realise that a collection of words of length *K* contains all the prefixes with *K*′<*K* (only the last *K*−*K*′ K-mers at the end of each sequence are lost). Regardless of word length, the number of words is practically the same (sequence length *L*−*K*+1; with *L*>>*K*). Therefore the dictionary is calculated only once using a large *K* value (*K*=32 by default). It is important to note that although *K* is calculated with a value of 32, the value of *K*′ is selected by the user at the seed points step, based on their knowledge of the sequences’ similarity.
2. Words are compressed on disk with a compression rate of 4 by using 2 bits per letter. This is possible because the K-mers are strictly composed of the {A, C, G, T} symbols of the DNA alphabet.
3. Larger *K* values produce a lower number of word matches between sequences, mainly due to less frequent repetitions, and result in a greatly decreased number of potential seed points from which to extend the alignment. On-the-fly dictionary analysis of stored words (repetitions, low complexity regions, etc.) help users select the most appropriate *K* value. In some circumstances low complexity regions (LCRs) can result in an excessive number of seed points or hits that can severely affect performance. GECKO includes a sampling procedure that limits the maximum number of hits analysed in a given area according to a user defined parameter. This effectively limits the number of hits in repetitive regions to a number of equally spaced “samples”, thus reducing the processing impact of LCRs without affecting normal sequences.
4. It is possible to further reduce the number of selected hits by using a proximity criterion, whereby additional seed points must be separated by a minimum distance parameter from other hits in order to be extended.
5. The computed K-word dictionaries remain available for subsequent processing when comparing genomes, which significantly reduces I/O load.

To reduce computational space usage we followed a similar strategy to that used by some existing solutions, which depends on the identification of common K-mers present in both sequences that are then used as seed points for local alignments.

### Modular design

As mentioned above, the second major improvement of our design was to modularise the process. The application is designed to be used for multiple genome data analysis, allowing for parameter sensitive studies as well as all-versus-all comparisons of genome collections. With the aim of reducing dependencies and repetitive actions, we organised the application workflow as follows (see Fig. 1):

1. One-off creation of a K-mer dictionary for each genome or sequence. The dictionary is stored on disk as a hash table, containing the words that appear in the sequence together with their positions.
2. Once calculated, K-mer dictionaries are then used to identify starting points (or hits) that will be used to obtain the HSPs. These seed points correspond to all possible matches produced between dictionary words. It is worth noting that the K value is parameterised at this point, with smaller K values derived as prefixes from the same dictionary.
3. Next, the application produces a local alignment (i.e. the HSPs) based on the calculated starting points, extending them in forward and reverse directions. From this point, all hits covered by a valid HSP are not analysed further.
4. To illustrate possible post-processing steps, several accessory modules have been developed such as HSP visualisation (equivalent to the Mummerplot application in the MUMmer suite); data format converters to allow the use of other visualisation software packages and further data analysis tools such as the K-mer frequency analysis program.

With minimal performance losses several software development features have been incorporated into GECKO to enable the development of a set of multi-platform applications. Examples include the usage of generic data types with the same representations in 32 and 64 bit architectures, the implementation of data access functions to read/write binary files in order to avoid Endianness problems and buffering strategies to minimise I/O operations and improve performance.

In the following sections we go into the details of each step performed by the GECKO application in chronological order.

#### Dictionary calculation

The dictionary calculation is based on the well-known binary tree in computer sciences. Each tree node contains a word (key) and its list of occurrences (values). Following the behaviour of a binary tree, left hand side nodes of a given tree come lexicographically before nodes on the right hand side. To avoid memory consumption problems caused by the huge number of possible words (i.e. a theoretical maximum of 4^*K*^ different words, without counting repetitions), we decided to split the calculation in *p* steps (with *p* being a multiple of 4), thus reducing the amount of memory used by the program by a factor of *p* (assuming a normal distribution of words). To split the dictionary and conserve its lexicographical order, a prefix of length log4*p* is used. This strategy requires us to iterate *p* times over the whole sequence, using a different lexicographically-organized prefix each time to preserve word order. To avoid memory allocation requests for each node, a single memory pool is reserved at the beginning of the process. New memory pools are then only reserved once the currently reserved memory is used up. To obtain the final result we traverse the tree in order, storing the word contained in the node together with the list of occurrences. We considered other strategies for this step, such as a prefix tree and a suffix array, but found that they experience memory consumption issues similar to the problems faced by existing software approaches.

#### Hits determination

The second section of the workflow corresponds to the identification of the starting points or seeds for the local alignment. If a word *w*_*i*_ appears *n* times in the first sequence at positions *p*_*j*_(*j*=1...*n*); and the same word *w*_*i*_ appears *m* times in the second sequence at positions *p*_*k*_(*k*=1...*m*), a hit will occur in all (*p*_*j*_,*p*_*k*_) coordinates producing the following set *h*={(1,1),...(1,*m*),(2,1),...(2,*m*),(*n*,1),...(*n*,*m*)}. All these hits are then considered starting positions for possible local alignments. Depending on how similar the sequences are and also on the K value used, the number of resulting hits could be very high. It is highly recommended to mask low complexity regions in order to reduce the hits produced by repetitive sequences. To reduce the number of hits further we have applied a proximity approach, by which those hits on the same diagonal, defined as *d*=(*p*_*j*_−*p*_*k*_), and at a predefined distance are combined. This can be achieved quickly and easily by sorting hits by diagonal (and offset), what is performed using a threaded version of the quicksort algorithm, and then combining the hits that are within the distance parameter value.

#### HSP detection

The last calculation step consists of producing a set of ungapped HSPs that conform to a local alignment. An HSP is defined as a substring matching sequence whose positive accumulated score cannot be increased by extending the fragment at either of its extremes (i.e. until it attains a local similarity maximum between sequences). The score is calculated either by adding or subtracting a given weight value (usually on the basis of DNA identity) depending on if a match or mismatch is given, respectively. The fragment starts from a hit with a positive score (the seed points identified in the previous section), and is extended along the sequence modifying the overall HSP score until it becomes negative or the end of one of the sequences is reached (or both simultaneously). Fragment boundaries are positions that give the highest accumulated score at both ends as HSPs are extended in both directions along the sequence (forward and backward). The algorithm continues searching for HSPs within the next hit in the diagonal or the first one of the next diagonal. If the next hit in the same diagonal has been covered by extension of the previous HSP, it would not be used because it will result in a redundant sub-HSP within the previous one. GECKO outputs a set of identified HSPs that are defined by starting and ending coordinates in both sequences, together with HSP length, score and identity levels.

#### HSP post-processing

Almost all existing methods provide a way of graphically representing local alignments after computation. GECKO incorporates its own visualisation procedure that generates a PNG file as well as the ability to output its analyses in formats that can be processed by the visualisation methods included with existing analysis programs. In addition, GECKO includes post-processing applications that enable tasks such as the ability to apply additional filters to HSP collections or generate gapped alignment constructions based on ungapped ones.

## Results

### Dataset

The selected test dataset contains sequences of different sizes in order to thoroughly compare GECKO with other state-of-the-art methods under both favourable (large sequences) and unfavourable (short sequences) situations. Specifically, the dataset is composed of short (virus), medium (bacteria and fly), and large (mammalian) sequences (see Table 1 for sequence names and their GenBank accession numbers). The large mammalian sequences will also be used for an all-versus-all experiment.

**Table 1:** Dataset information. From left to right: Type of comparison for which the sequence is going to be used, species name, strand and/or chromosome of origin, GenBank accession number and size in Mbp

Test type	Species	Strain / Chromosome	Accession number	Mbp
Pairwise comparison	Tomato Yellow Leaf Curl Virus	TYLCV	GenBank:AM409201.1	0.004
	Tomato Yellow Leaf Curl Virus	TYLCV-lr2	GenBank:EU085423.2	0.004
	Buchnera aphidicola	APS (Acyrthosiphon pisum)	GenBank:NC_002528.1	0.636
	Buchnera aphidicola	5A (Acyrthosiphon pisum)	GenBank:NC_011833.1	0.640
	Escherichia coli	K-12	GenBank:NC_000913.2	4.596
	Escherichia coli	O157:H7 Sakai	GenBank:NC_002695.1	5.448
	Drosophila melanogaster	chromosome 2R	GenBank:NT_033778.3	20.948
	Drosophila pseudoobscura	strain MV2–25 chromsome 3	GenBank:NC_009006.2	19.604
Multiple comparison	Homo sapiens	chromosome 1	GenBank:NC_000001.11	246.600
	Pan troglodytes	chromosome 1	GenBank:NC_006468.3	226.172
	Macaca mulata	chromosome 1	GenBank:NC_007858.1	226.092
	Pongo abelii	chromosome 1	GenBank:NC_012591.1	227.768
	Gorilla gorilla	chromosome 1	GenBank:NC_018424.1	227.336
	Mus musculus	chromosome 1	GenBank:NC_000067.6	193.624
	Rattus norvegicus	chromosome 1	GenBank:NC_005100.3	287.344
	Bos taurus breed Hereford	chromosome 1	GenBank:AC_000158.1	156.840
	Canis lupus familiaris breed Boxer	chromosome 1	GenBank:NC_006583.3	121.516
	Sus scrofa breed mixed	chromosome 1	GenBank:NC_010443.4	312.336

### Infrastructure and reference software

GECKO performance will be compared against equivalent state-of-the-art applications such as Gepard [6], MUMmer [5], Mauve [17], LASTZ [18] and LAST [19–21]. Either the source code or pre-compiled binaries were downloaded from the sources provided in the corresponding manuscripts. GECKO was compiled using GNU C Compiler (GCC) version 4.8.2, with “-O3” and “-D_FILE_OFFSET_BITS=64” compiling options (in the same way reference software packages were compiled). All the reference software was used in their command line versions in order to do a fair comparison with GECKO which is also executed through the command line (more details about execution parameters in the Section 3.3 of the Additional file 1).

The tests reported in this document were performed using an Openstack cloud instance configured with 4 Intel Xeon E312xx (Sandy Bridge) 2.0GHz equivalent cores, 8GB of RAM and the Ubuntu 12.04 LTS 64-bit operating system. For storage, a 300GB Openstack volume was used. The underlying physical disks of the Openstack setup were conventional ones (500GB, 16MB buffer, SATA 3, 7200 RPM). The cloud instance was deployed within the RISC Software GmbH cloud facilities in Hagenberg, Austria. Due to the inability of some current software to run in the mentioned infrastructure with large sequences (see the notes of Table 2), we additionally used Picasso shared memory multiprocessor located at the University of Málaga (Málaga, Spain). It contains 7 nodes, each with eight Intel E7-4870 processors which delivers 96 Gflop/s each, giving a peak performance of 5 Tflop/s. Each node has 2 TB of RAM giving an aggregate memory of 14 TB.

**Table 2:** Execution time in seconds for the comparison of the sequences listed in Table 1 under “pairwise comparison” (lowest execution time and memory consumption of each row are highlighted in bold). The comparison of mammalian chromosomes was also included to test the ability of GECKO and reference software packages to function when analysing very large datasets. The dictionary calculation time is included in the reported times, since the dictionary were not pre-calculated. “n.a.” indicates that resource problems prevented analysis execution and the presence of (*^1^) after some execution times indicates that the time was measured in a bigger machine because in such cases they were using more than 8GB of memory (more details of these cases in the Additional file 1 Section 3.3)

	Gepard		MUMmer		Mauve	
Comparison	Time	Memory	Time	Memory	Time	Memory
TYLCV-TYLCV-lr2	0.84	52824	**0.00**	2944	0.06	**2800**
BuchneraAPS-BuchneraBp	2.56	74808	**0.44**	**11100**	6.73	14304
E.colik12-E.coliO157	33.12	378412	10.63	**79212**	45.92	99880
D.Melanogaster-D.Pseudoobscura	238.34	716244	45.99	355272	294.92	379912
H.Sapiens-Chr1-P.Troglodytes-chr1	**7** **0** **8** **4** **.** **0** **0** ^∗1^	49788208	23226.00^∗1^	15747168	>604800.00	n.a.
	LASTZ		LAST		GECKO	
Comparison	Time	Memory	Time	Memory	Time	Memory
TYLCV-TYLCV-lr2	0.04	67388	**0.00**	3024	0.36	1564816
BuchneraAPS-BuchneraBp	0.46	71244	46.20	475912	1.60	1564816
E.colik12-E.coliO157	**1.83**	95884	109.00	1972028	17.20	1564816
D.Melanogaster-D.Pseudoobscura	**19.64**	**190448**	1593.00	5436716	48.72	1564816
H.Sapiens-Chr1-P.Troglodytes-chr1	78360.00	5782352	n.a.	312065840	**11848.15**	**1564816**

Results shown in this section (Table 2) correspond to sequential (one core) execution of each module except for the hit sorting method that used 8 threads running on one 4 core CPU. Further benchmarks using diverse collections of additional data are available in the Additional file 1.

### Pairwise tests

Multiple tests of the proposed out-of-core implementation have been designed within the simple pairwise comparison framework to evaluate memory consumption as a function of sequence length.

### Multiple comparison tests

**Fig. 2:**
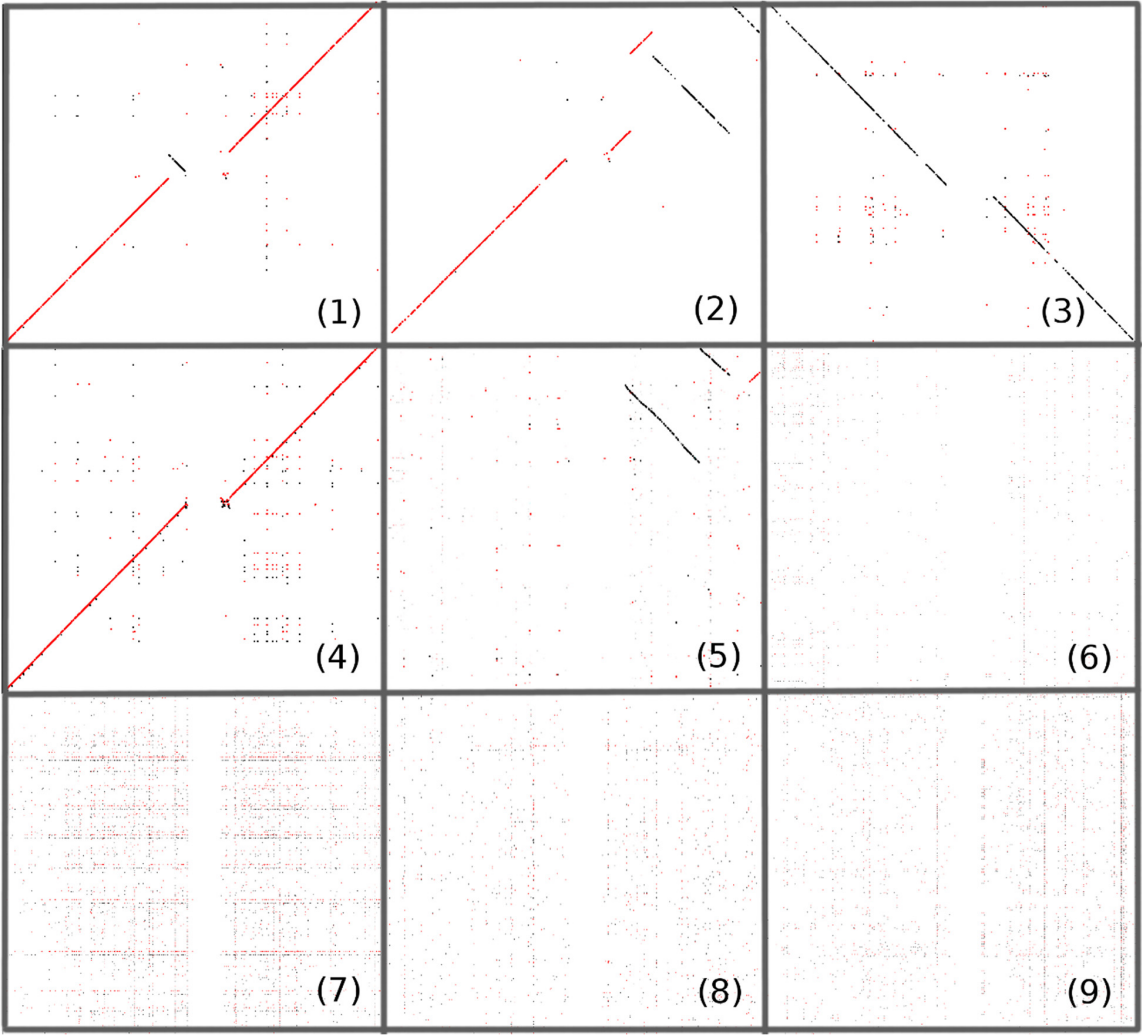
Separate dotplot-like representations of Human chromosome 1 (X-axis) compared to equivalent chromosomes from several other mammalian species: (**1**) Pan troglodytes, (**2**) Macaca mulata, (**3**) Pongo abelii, (**4**) Gorilla gorilla, (**5**) Mus musculus, (**6**) Rattus norvegicus, (**7**) Bos taurus, (**8**) Canis familiaris and (**9**) Sus scrofa. Red colour indicates forward strand fragments and black the reverse strand ones. Plots indicate that there are closely-related (from 1 to 5) and remotely-related (from 6 to 9) sequences. This is caused by the fact of that chromosome numbering was based on their length and not their content. For example, human chromosome 1 is present in several chromosomes of Bos Taurus (but not in the first chromosome, as can be deduced from sub-figure 7). An image with the first five sub-plots projected over one sequence is provided in the Additional file 1

This exercise was designed to test the advantages of saving intermediate results to disk. The test involves comparing human (Homo sapiens) chromosome 1 against the same chromosome in several other species. Figure 2 displays the visualisation of the resulting HSPs for *P. troglodytes, M. mulatta, P. abelii, G. gorilla, M. musculus, R. norvegicus, B. taurus, C. familiaris and S. scrofa.* It is worth noting, that only execution times of some methods are shown, due to the inability of the rest to run these tests in the mentioned infrastructure.

In all cases, we took into account the execution time of the full pipeline, as this test was designed to evaluate the worst-case situation. As explained in the Methods section, GECKO only needed to obtain the dictionaries once for the previous set of comparisons. For the sake of understanding the impact of this step and to aid comparisons with other methods, GECKO dictionary calculation times are shown in Table 3 and the total time is shown in Table 4.

**Table 3:** Dictionary calculation execution time in seconds for the sequences listed in Table 1 under the multiple comparison test type

Sequence (chr1)	Time
Homo sapiens (HS)	747.53
Pan troglodytes (PT)	630.07
Macaca mulata (MM)	649.26
Pongo abelii (PA)	628.81
Gorilla gorilla (GG)	712.68
Mus musculus (MMu)	537.45
Rattus norvegicus (RN)	857.28
Bos taurus breed Hereford (BT)	451.83
Canis lupus familiaris breed Boxer (CF)	293.36
Sus scrofa breed mixed (SS)	980.99

### Results quality

Although the performance aspects of GECKO’s design are crucial, the production of high quality results is equally important. In this section we explain how we evaluated the quality of the results produced by our algorithm versus the other applications using the same parameters. The rationale behind our evaluation was to compare the coverage of the HSPs detected by each algorithm. To avoid biases in the evaluation we decide to obtain a consensus set of reference HSPs. This set is composed of those HSPs reported by at least half of the reference algorithms. The HSPs produced by GECKO were then mapped over the reference HSPs and the percentage of coverage recorded as a measurement of result quality. This means that matching positions reported by the consensus HSP reference and not reported by GECKO will push down the quality and vice versa. There are more sophisticated ways of comparing the results, such as only considering coding regions, or by qualifying and weighting matches depending on sequence type or section. However, we decided not to use these methods as they can incorporate noise or biases into the evaluation.

Following this procedure, we performed quality evaluation on sets of both closely- and remotely-related sequences in order to thoroughly study the results of GECKO. In the case of closely-related sequences, our evaluation determined that GECKO detected 3 % more HSPs than the consensus set. Moreover, GECKO obtained a larger dataset while maintaining identity values over 65 %, thus representing the identification of additional statistically-significant HSPs. For both short and long remotely-related sequences, GECKO again obtained an average of 3 % more HSPs with identity values above 65 %. In addition to the coverage study, we also evaluated the identity values of the HSPs reported by GECKO compared to those of LASTZ. This test produced similar results for the two methods albeit with slightly better values reported by GECKO (details of this evaluation can be found in Sections 5.1, 5.2 and 5.3 of the Additional file 1).

### Visualisation

Strictly speaking GECKO is not intended for dotplot-like visualisation. However, we provide two alternatives: (1) two different programs able to generate 2D representations, one for single pairwise comparison results, capable of analysing forward and reverse HSPs (see Fig. 2); and the second for multiple comparisons whereby all comparisons are projected over one of the sequences selected as the reference. Obviously, any of the compared sequences can be used as the reference; (2) small plugins that allow GECKO results to be converted into formats compatible with commonly used visualisation methods.

## Discussion

Considering that GECKO’s implementation was designed primarily for very large sequence comparisons, it compares surprisingly well with the reference software packages when analysing short sequences. It is as fast as Gepard even when the dictionaries were not pre-calculated. Gepard reports 33 s for 5 Mbp genomes, compared with 17 s for our implementation. In the cases of MUMmer, LAST and LASTZ, our execution time was greater, due to the different strategy we are following compared to the suffix array indexing they are using, but still the difference is acceptable since the execution time is not that high. However, for longer sequences, our method strongly outperforms existing methods. GECKO needed less than 2 h in average to compare chromosome 1 from different species (all possessing more than 120 Mbp) against the 3 h and a half in average of Gepard and MUMmer and the 29 h of LASTZ (average values extracted from Table 4). Since all the reference software packages manage data structures in core memory, their good performance with short sequences was predictable, but this also means that their performance degrades as sequence size grows, entering into starvation when no more computational resources are available. This is due in part to the use of the Suffix Array data structure which in one side reduces the computational complexity but in the other increases the memory consumption up to 9 times the length of the input sequence in the most efficient implementations. For comparison purposes and to prove the mentioned Suffix Array memory consumption, we implemented a Suffix Array version of the program which significantly reduces the computation time compared with our actual dictionary strategy, but as mentioned is using more memory (more details can be seen in the Additional file 1 Section 2.2 “Alternative dictionary calculation using Suffix arrays”). The results of these comparisons are shown in Table 2. and more details are available in the Additional file 1 at Section 3.3.

GECKO’s implementation showed real-world performance gains ranging from 133 % versus Gepard for TYLCV comparison, to 3269 % versus LAST in the case of Drosophila comparison (see Table 2).

### In- or out-of-core implementations and modularity

Traditionally, bioinformatics programs, in common with conventional software development practices, are designed to perform calculations with the data loaded in main memory. This is in order to take advantage of the difference in access time between main and external memory, which is in the range of several orders of magnitude. However, the growth rate of available data has been even greater than the growth of the typical amount of RAM memory available. Although some specialised infrastructures offer TB quantities of RAM, such facilities are not yet routinely available to the global research community, while the quantity of available sequence data continues to spiral.

Clearly, in the era of Big Data it is increasingly impractical to keep all the data in core. Consequently there is a pressing need to re-design trusted software packages, as well as to develop brand new software strategies to tackle this problem. It is valid to exploit the particular data flow of each specific application, but generic solutions that can be applied to new problems as they emerge should ideally be the final target of developers. In this sense, our work here explores how both approaches can be combined to better exploit their advantages. The out-of-core implementation used in GECKO has the following advantages:

1. It removes any dependence on K-mer size, giving rise to the possibility of using small prefixes for short sequences and bigger values for larger ones. We have identified 32 as a maximum K value that gives the exact matches that are useful for this type of application, especially while comparing distantly-related sequences. Greater K values did not produce enough seed points for a meaningful comparison (even with chromosome or genome-sized datasets).
2. Working in disk allows word dictionaries computed by previous program instances to be preserved in secondary storage, thus reducing the time required for multiple comparison studies. As can be seen in Tables 3 and 4, the time saved by dictionary pre-calculation is around 65 % of total elapsed time for remotely related sequences and 7 % for closely related ones. For all-versus-all studies, with *n*∗(*n*−1)/2 comparisons, the time reduction is even greater since we save repeating dictionary-recalculation *n*−1 times. This is one of the drawbacks of current methods. It is important to note, that the time to access the dictionary from disk is less than the combined time to access the sequence from disk and re-build the dictionary, what confirms the improvement of storing it in disk.
3. The modular implementation of GECKO stores intermediate results to disk, which facilitates the development of small and simple software components that allow the exhaustive analysis of the program’s final output, as well as intermediate data such as word frequencies, word structure, comparative studies, extreme frequency analysis, functional genomics annotation and data visualisation. This method for organising execution even allows interactive analysis, with the possibility of re-executing specific parts of the analysis with different parameters.

**Table 4:** The numbers in the upper diagonal refer to the combined execution time for total HSP calculation, hit sorting and all-vs-all comparison of both strands (forward and reverse) in seconds (acronyms as described in Table 3). The charts in the bottom part are symmetric visual representations of the corresponding cell in the upper diagonal (bar colour legend: blue =GECKO; orange =Gepard; grey =Lastz; and yellow =MUMmer). The total execution time (in seconds) for all the comparisons were: GECKO - 318591, Gepard - 576889, Lastz - 4752315 and MUMmer - 558360. The total time for GECKO represents a dummy execution, the actual execution time (executing the dictionary calculation once) was of 142954

	Method	HS	PT	MM	PA	GG	MMU	RN	BT	CF	SS
HS	GECKO		19190	2438	11282	11433	9358	11367	3768	2944	5875
	Gepard		6152	2581	12973	2861	8644	11478	5850	5540	14880
	Lastz		158874	140255	117398	105912	108312	96593	63294	70904	157619
	MUMmer		13891	2536	7519	11083	31164	10566	2127	3277	10170
PT	GECKO			2932	10567	9287	9686	10800	3766	2939	5909
	Gepard			15662	22242	26400	27394	9113	11362	10445	12856
	Lastz			135093	191012	181386	210949	164312	171510	146315	115160
	MUMmer			6214	23434	43301	27051	9640	1594	2836	10384
MM	GECKO				3322	5432	5306	7558	4461	3294	6387
	Gepard				16356	16675	15573	9349	8663	7111	13517
	Lastz				141032	128324	136632	128874	72552	57393	133667
	MUMmer				4512	6669	17013	25387	1736	6005	10423
PA	GECKO					31137	10012	5907	3770	3081	5879
	Gepard					28282	25929	11305	11727	11680	14778
	Lastz					148768	167206	135357	82444	63000	157305
	MUMmer					15115	36458	19321	3330	3170	11564
GG	GECKO						9703	5957	5206	5294	7895
	Gepard						25819	9960	13355	13250	13244
	Lastz						137351	63414	44411	28089	66732
	MUMmer						36614	11729	6869	30429	45431
MMU	GECKO							5908	5159	5170	5873
	Gepard							10229	12219	11360	13493
	Lastz							58823	44641	32046	92761
	MUMmer							8546	1307	2756	10128
RN	GECKO								5930	5894	5935
	Gepard								8143	6458	17278
	Lastz								47869	39163	79895
	MUMmer								1979	4702	15277
BT	GECKO									3777	5914
	Gepard									6574	9827
	Lastz									21861	56029
	MUMmer									717	1608
CF	GECKO										5889
	Gepard										8302
	Lastz										51778
	MUMmer										2777
SS	GECKO										
	Gepard										
	Lastz										
	MUMmer										

### K-mer size parameter

It is not difficult to deduce from all of the above that the time needed to complete each analysis is determined by word size (K), and strongly affected by both noise and the algorithm’s seed point detection sensitivity. K-mers are stored as K = 32 to avoid having a large collection of dictionaries for each K value. K = 32 contains all the K-mers for *K*′<*K* with no additional processing, values that are especially useful to obtain enough exact matches for distant sequences. The software is designed such that it can be used with K values greater than 32 in case that future sequences and/or applications require such a change. Using an incorrect K value will degrade performance due to the large number of K-mers repetitions. To avoid starvation GECKO uses a sampling scheme for very common repetitions.

### Parallel execution

Although this work did not specifically address the issue of parallel execution, it is interesting to make some observations concerning this topic. Most of the processes described in the procedure are appropriate for parallel execution. A simple dataset-splitting process would allow the distribution of partial components from computation by different processors, followed by the collection and reassembly of results. For instance, it would be possible to distribute the processing of K-mers by the first program by sending words starting with a given prefix to different processors. Each process would produce a partial dictionary of words with a given prefix that can then be used by separate processes to calculate the seed points sharing the same prefix. For example, there are 64 different 3-letter prefixes, assuming a 4-letter DNA alphabet, which would produce 64 sub-dictionaries for each sequence and 64 comparisons to calculate seed points.

Although the processing times achieved by GECKO for the test analyses reported here were acceptable even when calculated using a single processor core, there are clear advantages to developing sequence analysis algorithms that take advantage of multi-core systems. In the context of ever increasing sequence dataset sizes, the development of parallel-processing implementations of sequence analysis software will be particularly important for multiple genome comparison studies.

## Conclusions

This document presents GECKO, a pairwise genome comparison application based on an enhanced reduction of memory consumption and computational space, combined with a modular out-of-core implementation with several important advantages, including K value independence, complexity reduction, high performance and high results accuracy.

Additionally, software components can be easily added to this application to extend its capabilities in the spirit of software developer collaboration. New modules can be added without needing any change to the current architecture. Example programs currently available include: K-mer frequency calculation, analysis of over- and under-represented word sets, pre-visualisation monitoring tools and full construction of local ungapped fragments including their alignment.

A set of benchmarks demonstrates the effectiveness of GECKO’s implementation, even on a single CPU machine.

GECKO does not require custom software or libraries to run. It can be executed within a variety of computing environments, from simple desktop PCs to more complex architectures such as clusters.

This software aims to facilitate massive comparisons of genome-sized sequences, as well as more complex evolutionary studies. Currently the output provided by this program is being used to identify evolutionary events such as inversions, transpositions and gene duplications. These studies have already provided new insights into evolutionary models of populations and species [22], as well as comparative metagenomic studies [23].

Ongoing work is focused on three main lines. The first is to develop additional modules to improve and extend the results generated by GECKO. The second is the parallelisation of the full pipeline and the last is to provide user-friendly environments on desktop and mobile platforms to make using GECKO as easy and accessible as possible.

## Additional file

Additional file 1 **Supplementary material.** (PDF 2160 kb)
