# Supplementary material for: Breaking the computational barriers of pairwise genome comparison
Source: BMC Bioinformatics. 2015 Aug 11;16(1):250. doi: 10.1186/s12859-015-0679-9 (PMC4531504; doi:10.1186/s12859-015-0679-9)
Supplement: Supplementary file 1 — Supplementary material. (PDF 2160 kb) [file 12859_2015_679_MOESM1_ESM.pdf]

Breaking computational barriers in pairwise genome comparison

Torreno, O., Trelles, O.

Supplementary material

Submitted to BMC Bioinformatics

Table of contents

1. Computational space reduction 3
  1.1. Words 3
  1.2. Hits 4
  1.3. Big-hits 5
2. Modular design 7
  2.1. The global idea 7
  2.2. First Step: Building the dictionaries 10
    Alternative dictionary calculation using Suffix arrays 12
  2.3. Second Step: alignments from hits 14
3. Benchmarking 16
  3.1. Dataset 16
  3.2. Reference software 18
  3.3. Execution time 19
    Determining the point where to start using GECKO 23
  3.4. Resulting dotplots 25
    TYLCV vs. TYLCV-lr2 25
    BuchneraAPS vs. Buchnera5A 26
    E.colik12 vs. E.coliO157 27
    D.Melanogaster-chr2R vs. D.Pseudoobscura-chr3 28
    H.Sapiens-chr1 vs. P.Troglodytes-chr1 29
    H.Sapiens-chr1 vs. (P.Troglodytes,M.mulata,P.Abelii,G.gorilla,M.musculus)-chr1 30
4. Programs usage 31
  4.1. Dictionary creation 31
  4.2. Hits 32
  4.3. SortHits 33
  4.4. FilterHits 34
  4.5. FragHits 38
  4.6. Additional programs 41
    readDict 41

alignFrags 42

5. Results quality 44

5.1. Closely related sequences 44

TLCV vs. TYLCV-lr2 44

Buchnera APS vs. Buchnera 5A 45

E.colik12 vs. E.coliO157 46

D. Melanogaster chr2R vs. D. Pseudoobscura chr3 49

5.2. Remotely related sequences 51

5.3. Study of the number of identities 54

5.4. Alignathon dataset study 58

1. Computational space reduction

1.1. Words

To reduce the computational space and accelerate data processing most of the proposed strategies uses some kind of pre-processing step. K-mers are used as prefixes for fast identification of matching words to be used as seed points from where to extend the local alignment.

```
seq : TCAGACGATT GAAGAATCAT n=20
pos : 0123456789 0123456789
```

A	2, 4, 7, 11, 12, 14, 15, 18	AA	11, 14	AAG	11
C	1, 5, 17	AC	4	AAT	14
G	3, 6, 10, 13	AG	2, 12	ACG	4
T	0, 8, 9, 16, 19	AT	7, 15, 18	AGA	2, 12
		CA	1, 17	ATC	15
		CC		ATT	7
		CG	5	CAG	1
		CT		CAT	17
		GA	3, 6, 10, 13	CGA	5
		GC		GAA	10, 13
		GG		GAC	3
		GT		GAT	6
		TA		TCA	0, 16
		TC	0, 16	TGA	9
		TG	9	TTG	8
		TT	8		

In the image a hash table using different "prefix" length (K=1, 2, 3). The header contains the "word" and the table contains the positions in which that word appears in the sequence. As longer the prefix is, the shorter the number of "occurrences" is. In this case, the hash is built-up by "full-identity", thus all the words are the same for a given header entry (this is a tradeoff between sensitivity and memory requirements).
The number of putative hash-headers is 4^K where K is the word length, however NOT all the combinations are present in the sequence. The exact number of words is L-K+1 being L the sequence length.

1.2. Hits

Hits are word matches to be used as seed points. The number of hits depends on the number of matching-word repetitions, and it depends also on the word size (K).

Example:

Hash Table for Seq X

Positions of the symbols

pos : 12345678901

seqX: TCAGACGATTG n=11

Hash Table (seqX for K=1)

A	3, 5, 8
C	2, 6
G	4, 7, 11
T	1, 9, 10

Hash Table for Seq Y

Positions of the symbols

pos : 1234567890

seqX: ATCGGAGCTG n=10

Hash Table (seqY for K=1)

A	1, 6
C	3, 8
G	4, 5, 7, 10
T	2, 9

Identical words produce hits in the coordinates they appear (diag= h - v, when xh matches yv)
(A) Produces hits in : (3, 1), (3,6), (5, 1), (5, 6), (8, 1) and (8,6)
(C) Produces hits in : (2, 3), (2, 8), (6, 3) and (6,8)

We would like to emphasise that although K is 32 for the dictionary step, the users can choose a different K value (e.g. K=1, 2, 3… 32) starting at the "hits determination" step based on their knowledge of the similarity shared by the sequences under comparison (higher numbers means less sensitivity and vice versa). We mention in the manuscript that all K'<K mers are detected, only as an observation. In fact, if we use K=32, the last K-mer in the sequence is in position L-32+1. Obviously, this last K-mer has 32 letters, therefore K-mers of length K=31, 30, … 1; are lost at sequence ends. However, this loss is minimal as the maximum fragment that could start at such a position would be no longer than 32 residues for obvious reasons.

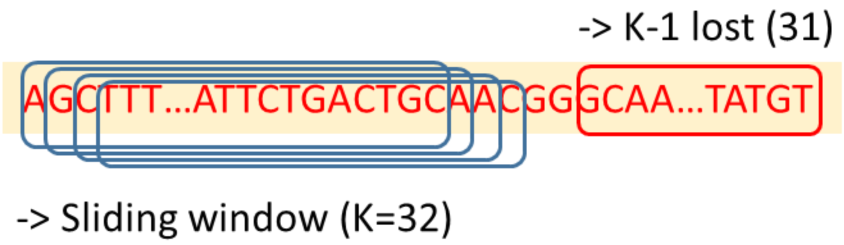

1.3. Big-hits

To reduce the computational space even further, the hits (i.e. matches of words coming from the sequences under comparison) are joined together based on their proximity.

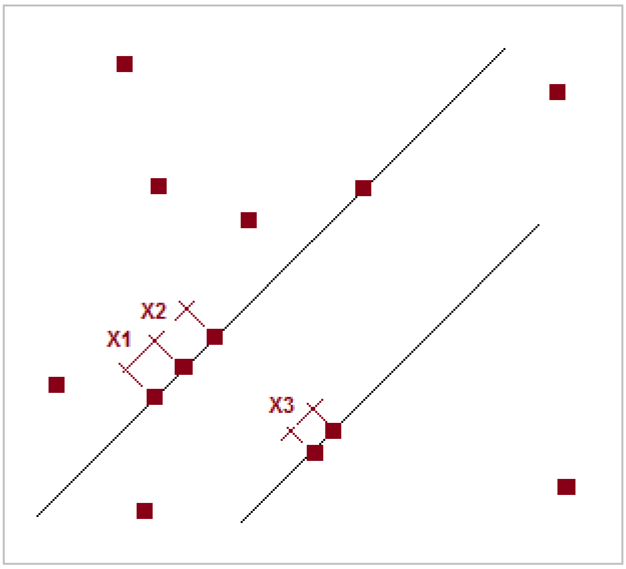

Hits in the same diagonal at a distance shorter that a parameter D will be joint to form a "big-Hit" (in the image the three hits in the first upper diagonal (at distances X1, X2 < D) and the two hits in the second diagonal (X3<D) are joint tor form a "3BigHit" and "2BigHit"

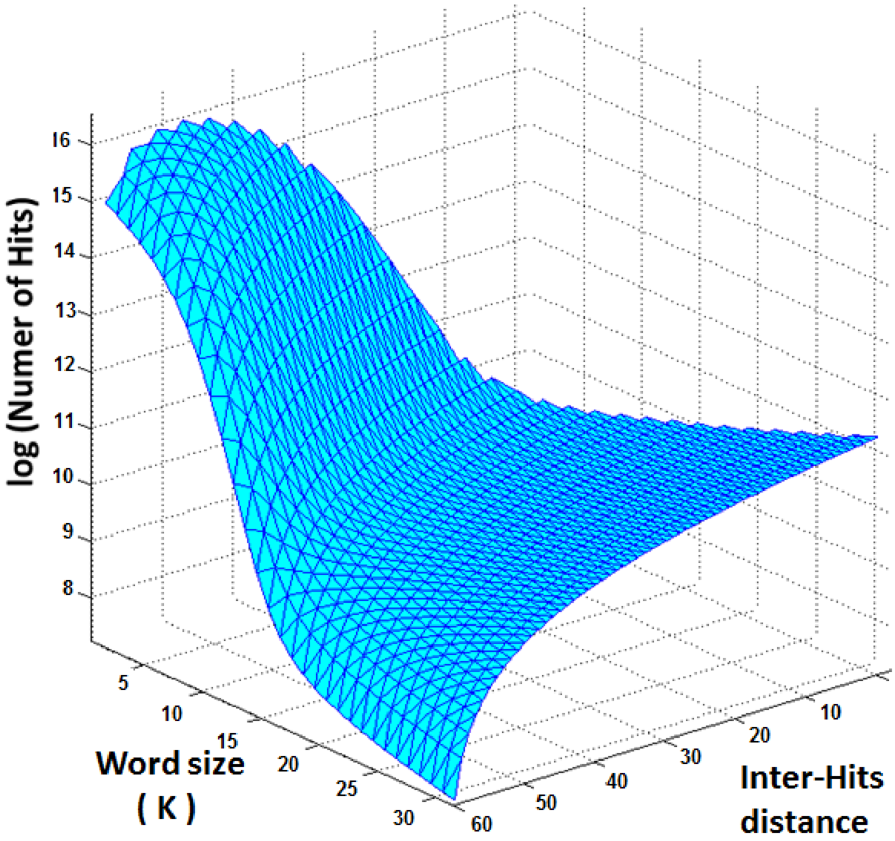

Behavior of the seed-points computational space as a function of K (word length) and the inter-hits distance parameter used to group neighbor hits. Real data (chromosomes X from several species) have been used in the simulation.

2. Modular design

2.1. The global idea

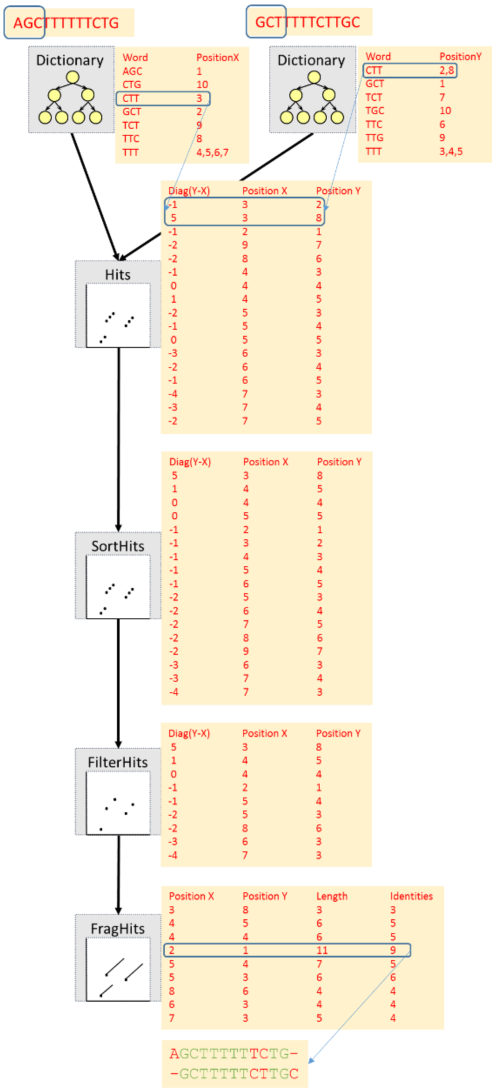

Pre-processing
Masking low complexity regions, …

Hashing
Including sub-processes such as sorting, grouping, etc.

HITS by diagonal
Including BigHits detection and pruning (see slide 8 for hits positions)

HITS Extension
Search for similarities using hits as seed points
Post-processing
Visualization, frequencies, words distributions, etc.

Figure 1 Dictionary function

```
function DICTIONARY(S,prefixSize)
    node ← tree
    for each prefix p in prefixes(prefixSize) do
        for each 32-mer(p) w in S do
            if w < node.key then
                INSERT(w, node.left)
            else if w > node.key then
                INSERT(w, node.right)
            else
                ADDREPETITION(w, node)
            end if
        end for
    end for
end function
```

Figure 2 Hits function

```
function HITS(dictS1,dictS2)
    while entries(dictS1)andentries(dictS1) do
        entryS1 ← dictS1
        entryS2 ← dictS2
        if entryS1 = entryS2 then
            positionsS1 ← POSITIONS(entryS1)
            positionsS2 ← POSITIONS(entryS2)
            PRODUCEHITS(positionsS1, positionsS2)
        else if entryS1 < entryS2 then
            entryS1 ← dictS1
        else
            entryS2 ← dictS2
        end if
    end while
end function
```

Figure 3 SortHits function

```
function SORTHITS(hits)
    for each hitChunk H in split(hits) do
        sortedHitsChunks ← QUICKSORT(H)
    end for
    MERGE(sortedHitsChunks)
end function
```

Figure 4 FilterHits function

```
function FILTERHITS(hits, distance)
    for each hits h1, h2 in hits do
        if h2.offset − h1.offset < distance then
            DELETE(h2)
        end if
    end for
end function
```

Figure 5 FragHits function

```
function FRAGHITS(hits, filteringParameters)
    for each hit h in hits do
        if h.offset > f.lastPosition then
            f ← EXTEND(h)
            if satisfy(f, filteringParameters) then
                SAVE(f)
            end if
        end if
    end for
end function
```

2.2. **First Step: Building the dictionaries**

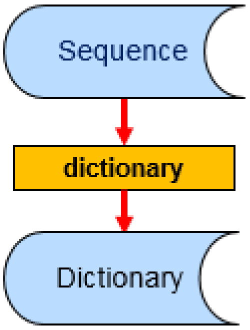

The dictionary calculation is based on the well-known binary tree in computer sciences. Each tree node contains a word (key) and its list of occurrences (values). Following the behaviour of a binary tree, left hand side nodes of a given tree come lexicographically before nodes on the right hand side. To avoid memory consumption problems caused by the huge number of possible words (i.e .a theoretical maximum of 4k different words, without counting repetitions), we decided to split the calculation in p steps (with

p being a multiple of 4), thus reducing the amount of memory used by the program by a factor of p (assuming a normal distribution of words). To split the dictionary and conserve its lexicographical order, a prefix of length log4p is used. This strategy requires us to iterate p times over the whole sequence, each time using a different prefix in lexicographical order to preserve word order. Another improvement is the reservation of a memory pool at the beginning of the process to avoid memory allocation requests occurring for each node. Instead, we request for a pool of memory and new memory pools are then only reserved once currently reserved memory is used up. To obtain the final result we traverse the tree in order, storing the word contained in the node together with the list of occurrences. We considered other strategies in this step, such as a prefix tree, but found that they experience memory consumption issues similar to the problems faced by existing software approaches.

Noteworthy to observe, this process needs to be done only once for each sequence (or twice if the complementary reverse is going to be also analyzed). The dictionary is computed for K=32 which contains all the prefixes for k'<K.

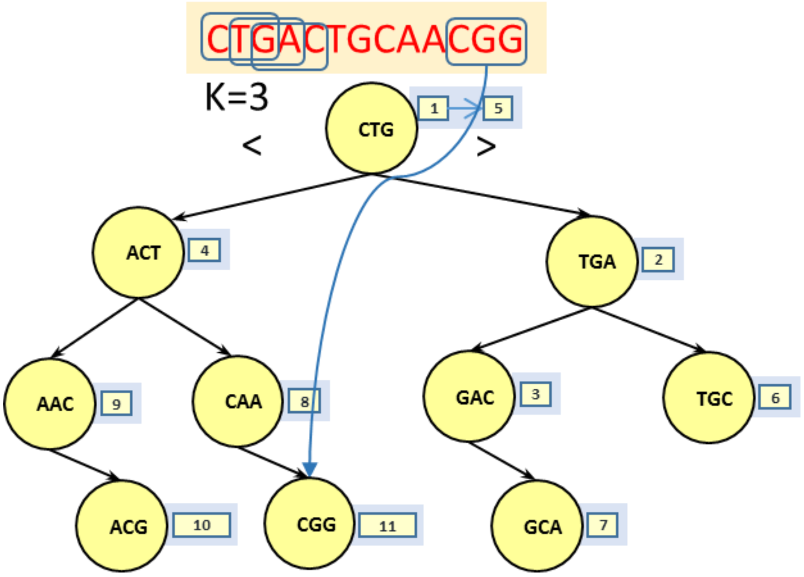

Visual representation of the binary tree used to store and sort K-mers. A sliding window is used to scan the sequence, then the word inside each window is inserted into the tree following lexicographic order. Blue boxes indicate the sequence position(s) of each word.

Alternative dictionary calculation using Suffix arrays

Most of the current methods to calculate genome comparisons/alignments (i.e. Gepard, Mauve, MUMmer, LASTZ) are using either Suffix trees or Suffix arrays as the indexing step in their algorithms.

In order to illustrate why we are using a customized solution instead of a Suffix array, we decided to implement a Suffix array version of our indexing step. This implementation confirmed what we can extract from the execution time and memory consumption data contained in the Results section of the paper. It is clearly faster than our customized solution for short sequences until a certain point where the performance gain starts to degrade. Besides execution time, the consumed memory of the suffix array implementation is of approximately 9 times the length of the input sequence what contrasts with our customized solution which has a constant consume of 1.5GB due to the buffering strategy we are using. As expected, the consumption for short sequences is lower than our solution because of the fixed buffer size, but as soon as the sequence length is big enough the consumption starts to be greater. In general the estimated consumption of 9 times the sequence length is acceptable for short sequences but for genomes longer than 1Gbp it starts to surpass the 8GB present in most of the current desktop machines.

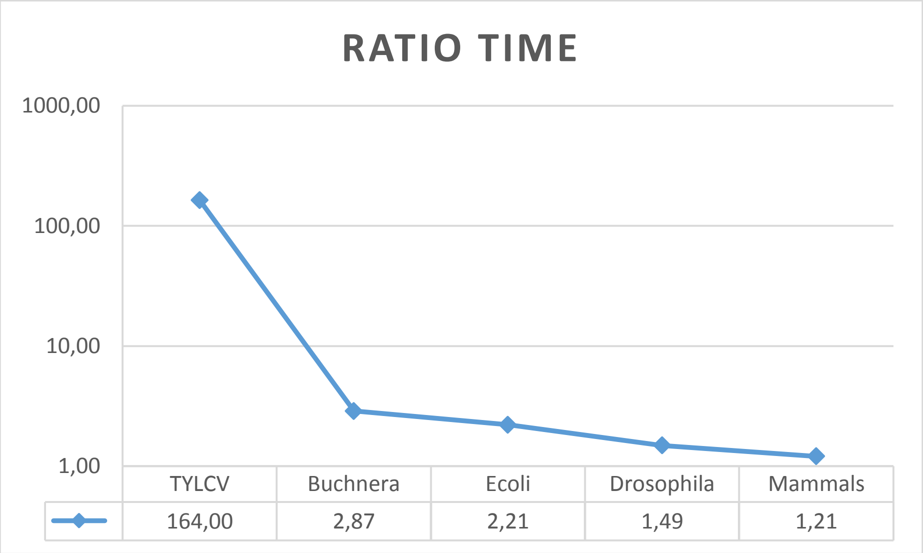

The previous figure confirms the fact that the execution time is getting closer between the two different implementations when the sequence size grows. This confirms that our customized solution, specifically designed for long sequences, has a comparable performance for long sequences.

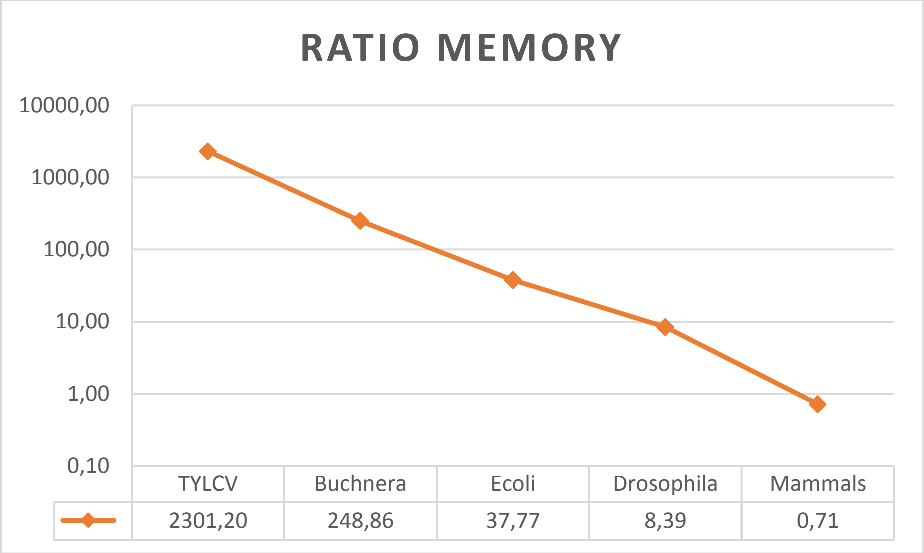

The previous figure confirms that for long sequences the memory consumption of our customized solution is lower than the one of suffix arrays, and the ratio will even get closer to zero when the sequence size grows (since our solution has a constant memory consumption).

Below the data from which we obtain the previous conclusions is shown.

	Dictionary		Hits		Dict+Hits	
	time	memory (kb)	time	memory (kb)	time	memory (kb)
TYLCV	00:00,00	1564816	00:00,16	391204	00:00,16	1564816
Buchnera	00:00,08	1564816	00:00,75	391204	00:00,83	1564816
Ecoli	00:12,00	1564816	00:08,44	391204	00:20,44	1564816
Drosophila	00:38,03	1564816	00:29,59	391204	01:07,62	1564816
Mammals	02:15,00	1564816	23:25,74	391204	25:40,74	1564816

	Suffix array		Hits		sarray+hits	
	time	memory (kb)	time	memory	time	memory (kb)
TYLCV	00:00,00	680	00:00,00	680	00:00,00	680
Buchnera	00:00,10	6288	00:00,19	6288	00:00,29	6288
Ecoli	00:01,08	41428	00:08,19	41428	00:09,27	41428
Drosophila	00:06,86	186520	00:38,64	186520	00:45,50	186520
Mammals	01:46,69	2188768	19:29,58	2188768	21:16,27	2188768

2.3. Second Step: alignments from hits

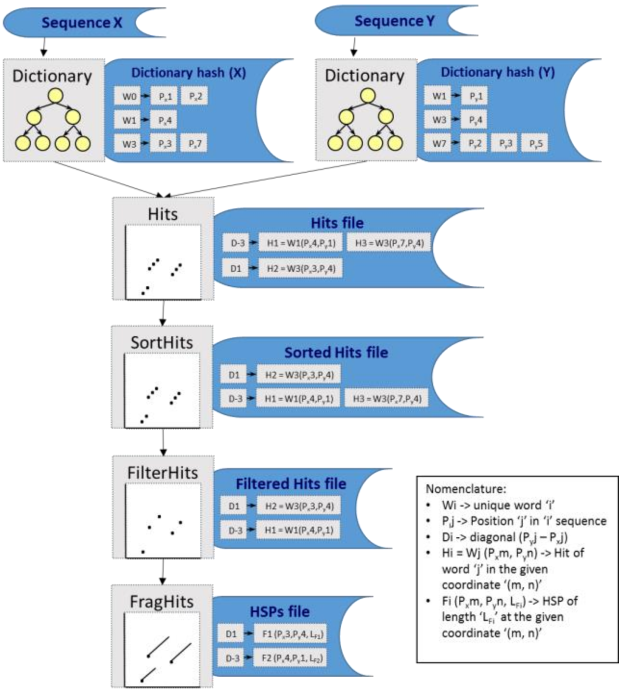

Starting: dictionaries of the sequences to compare

Hits: hits production based on identical words (the size of K can be redefined) to increse sensitivity

Sort: by diagonal and offset in the diagonal

Big-Hits: join hits by proximity

FragsFromHits:
The main procedure. Starting from seed-point extend the local ungapped alignment

Other: several tools can be used for postprocessing

Module	Description	Input/outputs
**Words**	Building a k-words dictionary.<br>- Words scanning<br>- Organize words in a hash table (disk). This table contains for each word the number of repeats and the positions of each repeat	Sequences /<br>Sequence dictionaries
**W2hd**	Organize words in a hash table (disk). This table contains for each word the number of repeats and the positions of each repeat	Ordered set of words /<br>2 levels hash table (Pfix
**Hits**	The same word in both seqs will produce a hit in each pair of position combinations. The diagonal number for the hit is also computed	Hash tables for each sequence /<br>Hits (diag, X,Y)
**BigHits**	Seeds identification<br>- Order the collection of hits (sortHits)<br>- Identify consecutive hits as big-hits<br>- [filtering of isolated hits]	Hits collection (diag, posX, posY) / Reduce Big-Hits collection (diag, posX, posY)
**Fragments**	Un-gapped fragment detection by extension of seed points	Big-Hits collection /<br>Un-gapped fragments
**Post-Process**	Post processing (available)<br>- Words frequencies<br>- Fragments distribution (Length, Score)<br>- Dotplot visualization<br>- Detailed fragment composition	Several of intermediate files /<br>Several outputs

3. Benchmarking

3.1. Dataset

Test type	Species	Strain / Chromosome	GenBank ACC	Mbp
Pairwise comparison	Tomato Yellow Leaf Curl Virus	TYLCV	AM409201.1	0.003
	Tomato Yellow Leaf Curl Virus	TYLCV-Ir2	EU085423.2	0.003
	Buchnera aphidicola	APS (Acyrthosiphon pisum)	NC_002528.1	0.620
	Buchnera aphidicola	5A (Acyrthosiphon pisum)	NC_011833.1	0.640
	Escherichia coli	K-12	NC_000913.2	4.500
	Escherichia coli	O157:H7 Sakai	NC_002695.1	5.400
	Drosophila melanogaster	chromosome 2R	NT_033778.3	21.000
	Drosophila pseudoobscura	strain MV2-25 chromsome 3	NC_009006.2	20.000
Multiple comparison	Homo sapiens	chromosome 1	NC_000001.11	241.000
	Pan troglodytes	chromosome 1	NC_006468.3	221.000
	Macaca mulata	chromosome 1	NC_007858.1	221.000
	Pongo abelii	chromosome 1	NC_012591.1	223.000
	Gorilla gorilla	chromosome 1	NC_018424.1	223.000
	Mus musculus	strain C57BL/6J – chromosome 1	NC_000067.6	190.000
	Rattus norvegicus	strain BN/SsNHsdMCW chromosome 1	NC_005100.3	281.000
	Bos taurus breed Hereford	chromosome 1	AC_000158.1	154.000
	Canis lupus familiaris breed Boxer	chromosome 1	NC_006583.3	119.000
	Sus scrofa breed mixed	chromosome 1	NC_010443.4	306.000

Table 1. Dataset information. From left to right: Type of comparison for which the sequence is going to be used, species name, strand and/or chromosome of origin, GenBank accession number and size in Mbp.

It is important to note, that although we are comparing the same chromosome of several sequences, the chromosome numbering depends on the sequence length rather than on the content. This is to say, that we will obtain significant alignments in a subset of comparisons, but not in all of them. This fact can be observed in the alignment of human with domestic cow contained below.

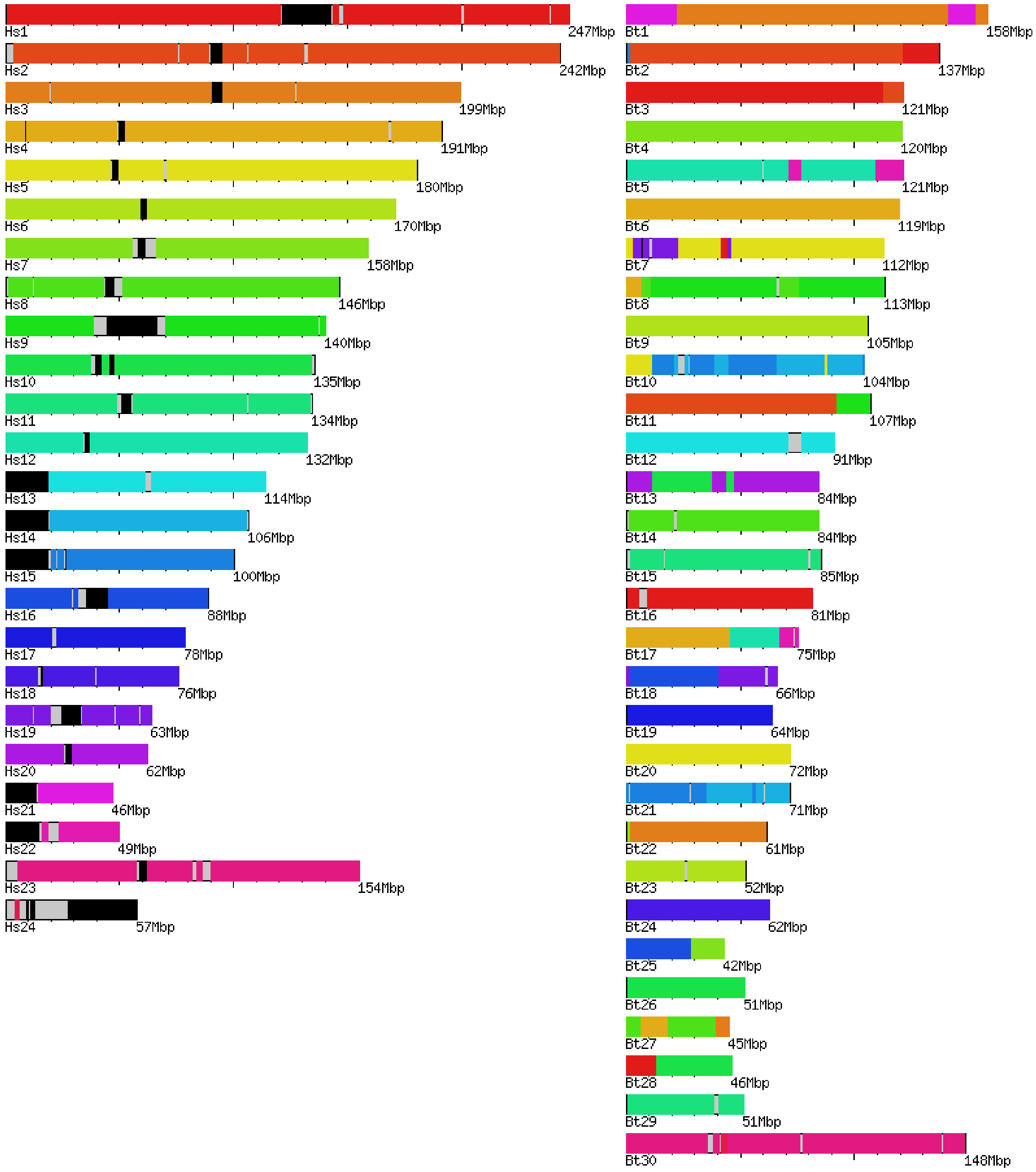

The previous figure is included to illustrate the non-presence of significant signals in some of the sub-plots of the new figure. In the previous figure, the human chromosomes are color-coded at the left hand side and at the right hand side are the

corresponding chromosomes of Bos Taurus. (Source: https://ccb.jhu.edu/bos_taurus_assembly.shtml, ftp://ftp.cbcb.umd.edu/pub/data/assembly/Bos_taurus/Bos_taurus_UMD_3.0/human_map/Hs-Bt.merge.map.png)

3.2. Reference software

The reference software used for comparison purposes are:

1. Gepard (version 1.30)
   In this case we used the command line version (gepardcmd.sh). An execution line example:

```
gepardcmd.sh -seq1 S1 -seq2 S2 -matrix matrix.mat -outfile plot.png
```

2. MUMmer (version 3.23)
   In this case we ask MUMmer to report all the matches (as GECKO is doing), because with the default options it is reporting only the maximal unique matches (MUMs). We ask MUMmer also to use compare only (A, C, G, T) with '-n' option and to do it with both sequence strands '-b'. An execution line example:

```
mummer -maxmatch -n -b –l 32 S1 S2
```

3. Mauve (version 2.3.1)
   In this case we did not use Mauve graphical user interface but the command-line program progressiveMauve with '—mums' to report the maximal unique matches as MUMmer is doing, '—skip-gapped-alignment' because GECKO is calculating ungapped HSPs and also specifying the score matrix. An execution line example:

```
progressiveMauve --mums --skip-gapped-alignment --substitution-matrix=matrix.mat --output=mauve.txt  S1 S2
```

4. LASTZ (version 1.02.00)
   In this case we ask LASTZ to use overlapping words during the search ('—step=1'), also to calculate ungapped HSPs ('--nogapped') and everything in both strands ('—strand=both'). An execution line example:

```
lastz S1 S2 --step=1 --nogapped --strand=both --format=maf --scores=matrix.mat
```

5. LAST (version 545)
   LAST first calculates a database (similar step to the one GECKO is performing) with the following command:

```
lastdb S1DB S1
```

   And then it performs the comparison. Specific parameters change the maximum number of initial matches per query position (-m 1000) in order to make it comparable to the limit of also 1000 used in GECKO. It was executed using the same scoring scheme ('-r' and '-q'), for both strands ('-s 2'),

calculating local alignment ('-T 0'), with a minimum initial match of 32 ('-l') and with an overlapping window along the query sequence of step 1 ('-k', similar to the '–step=1' of LASTZ). These specific parameters were used to obtain a fair comparison. An execution line example:

```
lastal S1DB S2 -m 1000 -r 4 -q 4 -s 2 -T 0 -l 32 -k 1
```

All the programs are using the following scoring matrix (or equivalent match/mismatch parameters in the cases they are not accepting a scoring matrix parameter):

```
     A     C     G     T
A    4    -4    -4    -4
C   -4     4    -4    -4
G   -4    -4     4    -4
T   -4    -4    -4     4
```

3.3. Execution time

	Gepard		MUMmer		Mauve	
Comparison	Time	Memory	Time	Memory	Time	Memory
TYLCV-TYLCV-Ir2	0.84	52824	**0.00**	2944	0.06	**2800**
BuchneraAPS-BuchneraBp	2.56	74808	**0.44**	**11100**	6.73	14304
E.colik12-E.coliO157	33.12	378412	10.63	**79212**	45.92	99880
D.Melanogaster-D.Pseudoobscura	238.34	716244	45.99	355272	294.92	379912
H.Sapiens-Chr1-P.Troglodytes-chr1	**7084.00***[1]	49788208	23226.00*[1]	15747168	>604800.00	n.a.

	LASTZ		LAST		GECKO	
Comparison	Time	Memory	Time	Memory	Time	Memory
TYLCV-TYLCV-Ir2	0.04	67388	**0.00**	3024	0.36	1564816
BuchneraAPS-BuchneraBp	0.46	71244	46.20	475912	1.60	1564816
E.colik12-E.coliO157	**1.83**	95884	109.00	1972028	17.20	1564816
D.Melanogaster-D.Pseudoobscura	**19.64**	**190448**	1593.00	5436716	48.72	1564816
H.Sapiens-Chr1-P.Troglodytes-chr1	78360.00	5782352	n.a.	312065840	**11848.15**	**1564816**

The execution time in the case of Gepard contains "*" because the program suffered memory consumption problems in our test infrastructure. This does not correspond in with what they report, see the table below extracted from: "Krumsiek, Jan, et al. (2007); "Gepard: a rapid and sensitive tool for creating dotplots on genome scale"; Bioinformatics Vol. 23 no. 8"

Sequence length	DOTTER	Gepard	Gepard pre-SA
10 000 bp	2 s	<1 s	<1 s
50 000 bp	30 s	<1 s	<1 s
100 000 bp	2 min 4 s	<1 s	<1 s
1 000 000 bp	2 h 10 min	5 s	4 s
5 000 000 bp	52 h 38 min[a]	47 s	40 s
Human chrom. I	382 years[a]	61 min	53 min

We confirmed this by executing Gepard in a bigger machine. The execution reported the use of almost 50GB of memory and a total time of 1 hour, 58 minutes and 4 seconds. We think the difference in execution time with GECKO resides in the fact that Gepard is not reporting a list of the resulting HSPs (which in this case is a big file which takes some time to be written) but a resulting 'png' image which provides a visual overview of the similarities shared by the sequences under comparison.

```
/usr/bin/time -v
/mnt/home/users/tic_182_uma/oscart/distantSequences/gepar
d-1.30/gepardcmd.sh -seq1 S1 -seq2 S2 -matrix
/mnt/home/users/tic_182_uma/os
cart/distantSequences/gepard-1.30/matrices/edna.mat -
outfile plot.png -lower 1000
Loading substitution matrix...
Loading sequence from S1
Loading sequence from S2
Calculating suffix array...
Calculating dotplot...
Creating image and writing to file...
      Command being timed:
"/mnt/home/users/tic_182_uma/oscart/distantSequences/gepa
rd-1.30/gepardcmd.sh -seq1 S1 -seq2 S2 -matrix
/mnt/home/users/t
ic_182_uma/oscart/distantSequences/gepard-
1.30/matrices/edna.mat -outfile plot.png -lower 1000"
      User time (seconds): 7306.41
      System time (seconds): 66.64
      Percent of CPU this job got: 104%
      Elapsed (wall clock) time (h:mm:ss or m:ss): 1:58:04
      Average shared text size (kbytes): 0
      Average unshared data size (kbytes): 0
      Average stack size (kbytes): 0
      Average total size (kbytes): 0
      Maximum resident set size (kbytes): 49788208
      Average resident set size (kbytes): 0
      Major (requiring I/O) page faults: 11
      Minor (reclaiming a frame) page faults: 35901
      Voluntary context switches: 193348
      Involuntary context switches: 1867492
      Swaps: 0
      File system inputs: 4648
      File system outputs: 5360
      Socket messages sent: 0
      Socket messages received: 0
      Signals delivered: 0
      Page size (bytes): 4096
      Exit status: 0
```

In the case of Mummer, the table contains “*” because it also suffered memory consumption problems in our 8GB infrastructure. We confirmed this fact with a later execution in a bigger machine (see the 15GB Maximum resident size highlighted in bold below). Anyway the execution time is greater than the one reported by GECKO (6 hours, 27 minutes and 6 seconds compared to 3 hours, 17 minutes and 24 seconds calculated as dictionary calculation time plus comparison time of GECKO). The output of the execution is:

```
/usr/bin/time -v mummer -maxmatch -n -b S1 S2 > mummer.mums
# reading input file "S1" of length 234708820
# construct suffix tree for sequence of length 234708820
# (maximum reference length is 536870908)
# (maximum query length is 4294967295)
# process 2347088 characters per dot
#.........................................................
.........................................
# CONSTRUCTIONTIME mummer S1 322.15
# reading input file "S2" of length 231158085
# matching query-file "S2"
# against subject-file "S1"
# COMPLETETIME mummer S1 21197.13
# SPACE mummer S1 450.64
      Command being timed: "mummer -maxmatch -n -b S1 S2"
      User time (seconds): 20777.10
      System time (seconds): 420.03
      Percent of CPU this job got: 91%
      Elapsed (wall clock) time (h:mm:ss or m:ss): 6:27:06
      Average shared text size (kbytes): 0
      Average unshared data size (kbytes): 0
      Average stack size (kbytes): 0
      Average total size (kbytes): 0
      Maximum resident set size (kbytes): 15747168
      Average resident set size (kbytes): 0
      Major (requiring I/O) page faults: 11
      Minor (reclaiming a frame) page faults: 238952
      Voluntary context switches: 88218
      Involuntary context switches: 2751913
      Swaps: 0
      File system inputs: 0
      File system outputs: 213119008
      Socket messages sent: 0
      Socket messages received: 0
      Signals delivered: 0
      Page size (bytes): 4096
      Exit status: 0
```

In the case of LAST, the table contains "n.a." because it suffered memory consumptions problems in our 8GB infrastructure. In this case the execution was even not possible in a bigger machine (see the more than 300GB used in the maximum resident size highlighted in bold below). This is in contrast with the statement they make in their webpage (http://last.cbrc.jp): "Human vs. mouse. This took about 1 day on 1 CPU, and less than 2 GB of RAM." We believe it is because of the parameters we are using, but in order to do an apples-to-apples comparison with GECKO the parameters need to be fixed to such values. In any case, the execution time reported until it crashed it is much greater compared to the one of GECKO (39 hours 57 minutes and 57 seconds in LAST compared to 3 hours, 17 minutes and 24 seconds calculated as dictionary calculation time plus comparison time of GECKO).

```
/usr/bin/time -v ~/bin/lastdb S1DB S1
      Command                 being                 timed:
"/mnt/home/users/tic_182_uma/oscart/bin/lastdb S1DB S1"
      User time (seconds): 1883.72
      System time (seconds): 6.96
      Percent of CPU this job got: 98%
      Elapsed (wall clock) time (h:mm:ss or m:ss): 31:56.63
      Average shared text size (kbytes): 0
      Average unshared data size (kbytes): 0
      Average stack size (kbytes): 0
      Average total size (kbytes): 0
      Maximum resident set size (kbytes): 5554832
      Average resident set size (kbytes): 0
      Major (requiring I/O) page faults: 22
      Minor (reclaiming a frame) page faults: 15134
      Voluntary context switches: 1174
      Involuntary context switches: 164964
      Swaps: 0
      File system inputs: 0
      File system outputs: 2665792
      Socket messages sent: 0
      Socket messages received: 0
      Signals delivered: 0
      Page size (bytes): 4096
      Exit status: 0
/usr/bin/time -v ~/bin/lastal S1DB S2 -m 1000 -r 4 -q 4 -
s 2 -T 0 -l 32 -k 1 > last.maf
lastal: out of memory
Command exited with non-zero status 1
      Command                 being                 timed:
"/mnt/home/users/tic_182_uma/oscart/bin/lastal S1DB S2 -m
1000 -r 4 -q 4 -s 2 -T 0 -l 32 -k 1"
      User time (seconds): 78617.06
      System time (seconds): 50773.25
      Percent of CPU this job got: 91%
```

```
Elapsed (wall clock) time (h:mm:ss or m:ss): 39:26:01
Average shared text size (kbytes): 0
Average unshared data size (kbytes): 0
Average stack size (kbytes): 0
Average total size (kbytes): 0
Maximum resident set size (kbytes): 312065840
Average resident set size (kbytes): 0
Major (requiring I/O) page faults: 116838
Minor (reclaiming a frame) page faults: 15271802
Voluntary context switches: 13365888
Involuntary context switches: 13713301
Swaps: 0
File system inputs: 0
File system outputs: 127320
Socket messages sent: 0
Socket messages received: 0
Signals delivered: 0
Page size (bytes): 4096
Exit status: 1
```

In the case of Mauve (i.e. progressiveMauve) in the table appears “>604800.00” what means that the execution time is greater than a week (the maximum execution time of a job in our bigger system). Since the job was cancelled we did not obtain the memory consumption, that is why the table contains “n.a.”.

Determining the point where to start using GECKO

The following chart compares the execution times of GECKO and MUMmer showing that MUMmer is significantly faster when analysing short sequences. However, since the difference between the processing of smaller sequence datasets (< 5 Mbp) is in the range of seconds, it may not be worth switching between the two different methods for this reason alone. If choosing an analysis method for speed, the execution time for GECKO and MUMmer is similar on *Drosophila* genome-sized sequences (around 21 Mbp), and we would suggest GECKO as the better choice from this point onwards. However, there are other reasons to choose GECKO, for example MUMmer only reports the occurrence position in both sequences plus the length of the alignment, in contrast to the default GECKO output, which includes additional useful values such as the number of identities, score, similarity, etc.

In the end, the choice of which software to use depends on many variables such as the type of analysis or the species being worked on. However, there is a clear trend to think in terms of big-sequence analysis, not only going from genes to genomes but also from pairwise comparison to multiple genome comparison. For a lab that plans to look

at more complex sequence analysis, we truly believe that the way GECKO avoids multiple dictionary re-calculations, or hits computation while filtering fragments with different parameters, makes it the best option in many, if not most, situations.

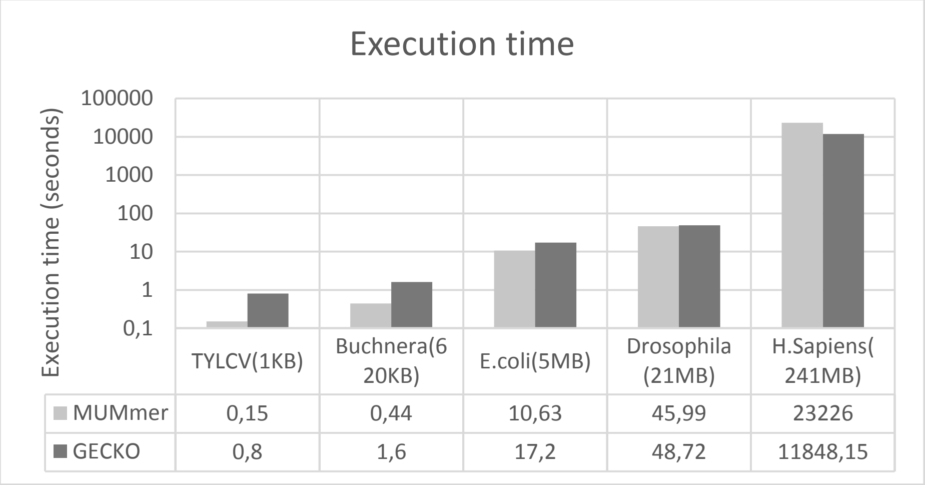

3.4. Resulting dotplots

TYLCV vs. TYLCV-lr2

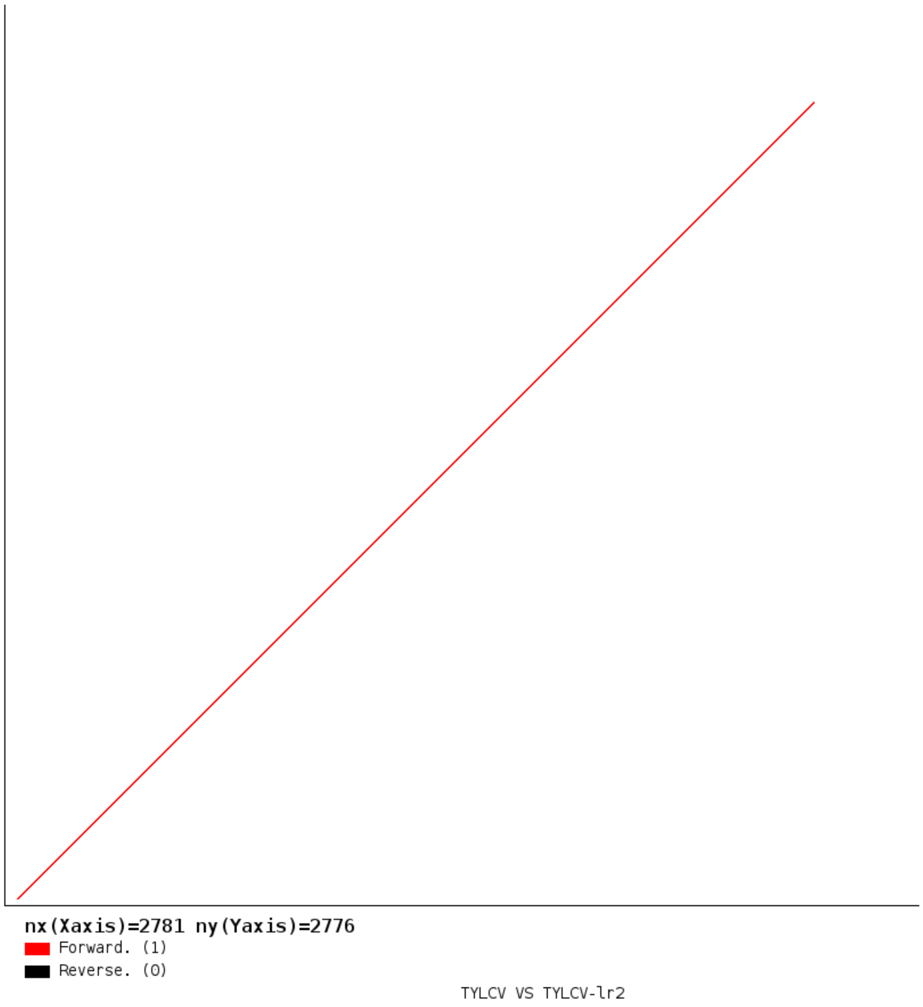

BuchneraAPS vs. Buchnera5A

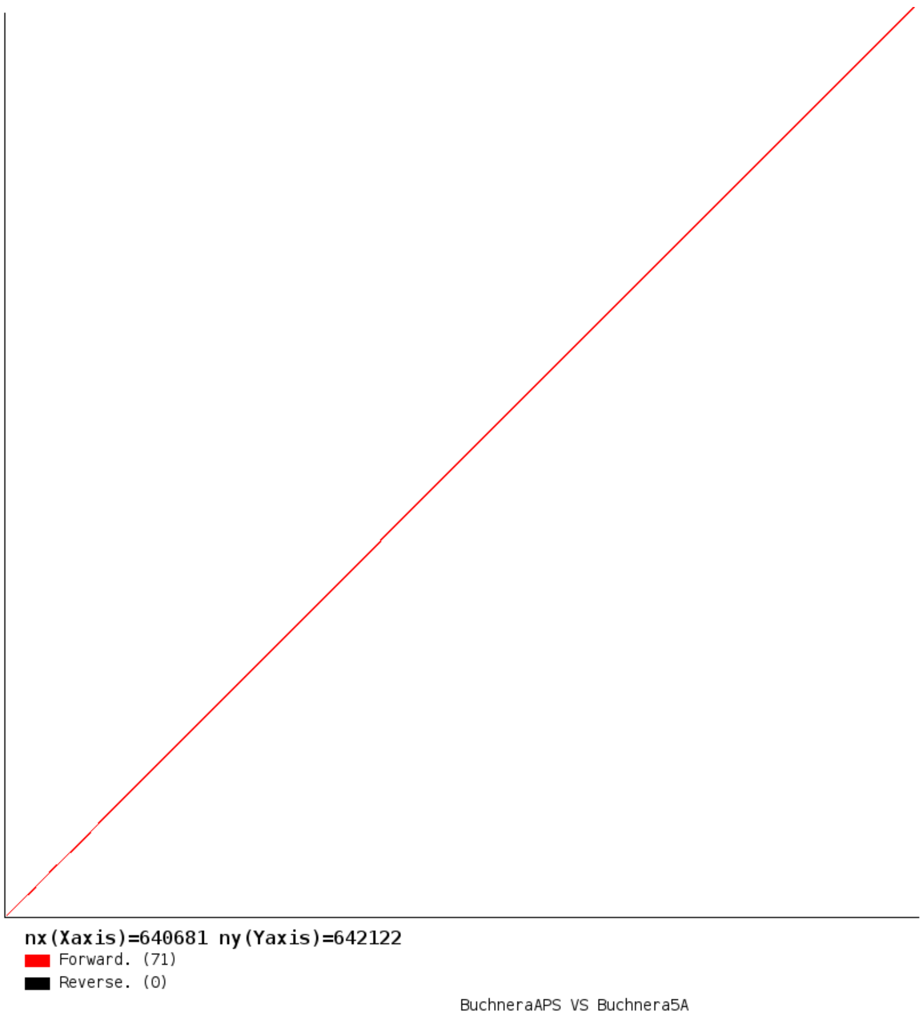

E.colik12 vs. E.coliO157

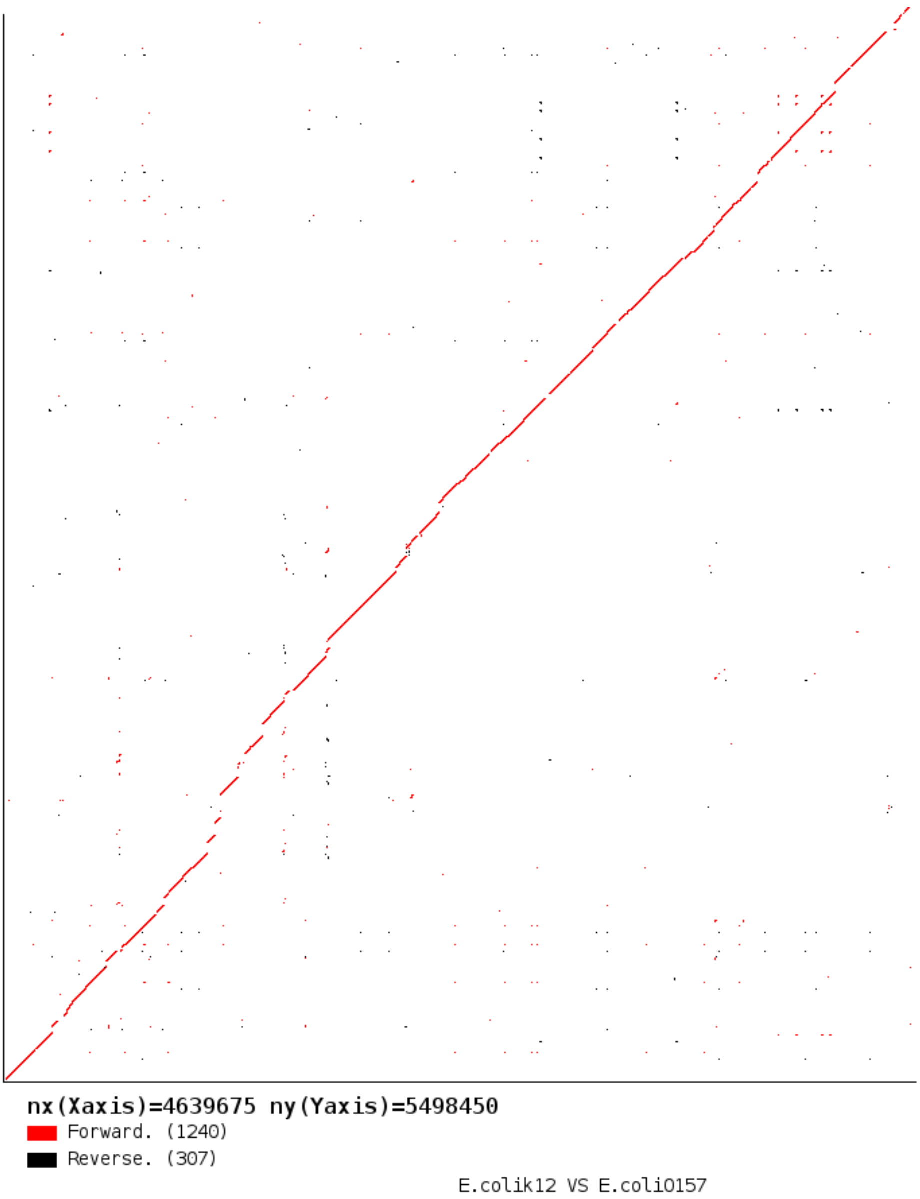

D.Melanogaster-chr2R vs. D.Pseudoobscura-chr3

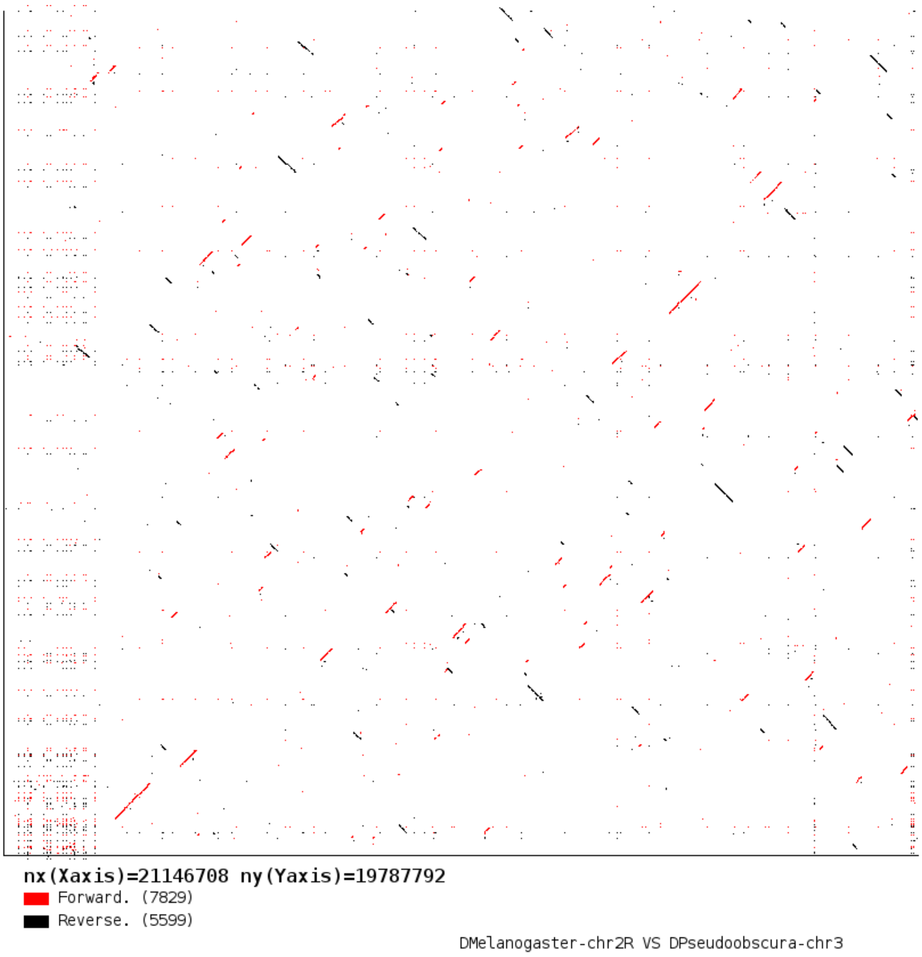

H.Sapiens-chr1 vs. P.Troglodytes-chr1

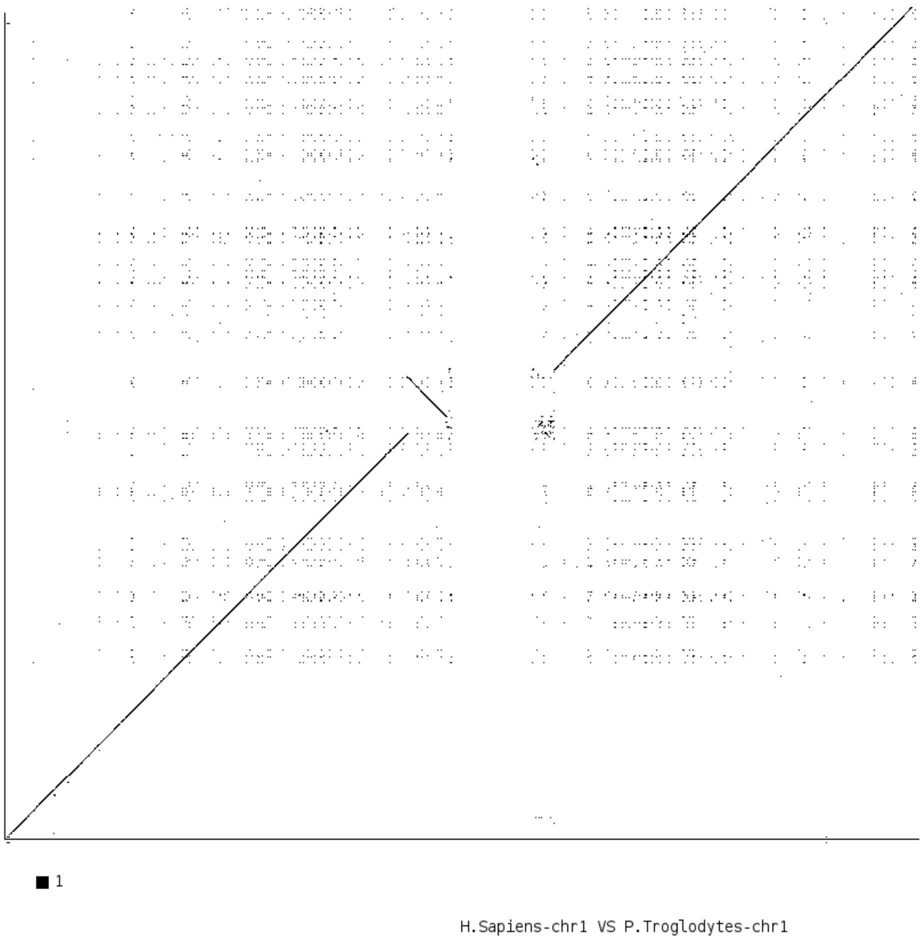

H.Sapiens-chr1 vs. (P.Troglodytes,M.mulata,P.Abelii,G.gorilla,M.musculus)-chr1

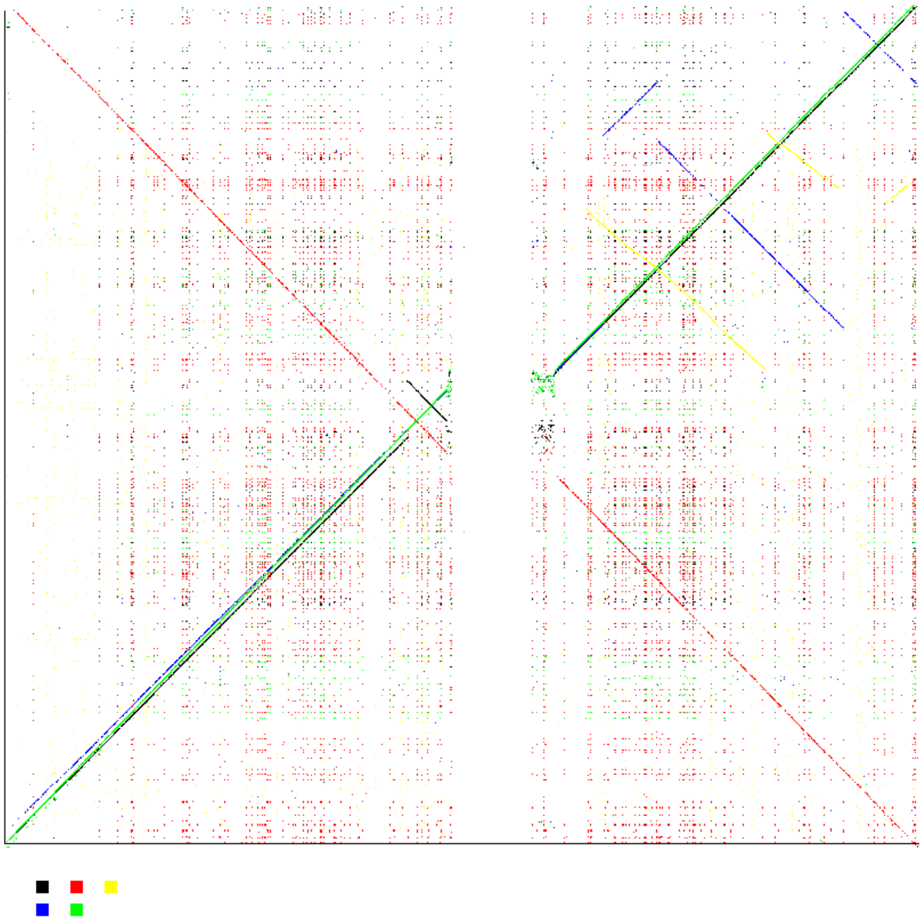

4. Programs usage

This is a basic usage explanation, a guided is available at (http://chirimoyo.ac.uma.es/gecko/documents/GuidedExercise-fromGENOMES2Visualization-reduced-v0.1.pdf).

4.1. Dictionary creation

```
Usage: dictionary seq.fasta prefixSize prefixOutfile
```

Parameters:

- Seq.fasta: input sequence from which the words to be stored in the dictionary will be extracted.
- prefixSize: this parameters indicate the number of iterations that the program will do. The number of iterations is 4^p. Example with p=1 the first iteration will look for the words starting by 'A', the second iteration the words starting with 'C', the third with 'G' and the fourth with 'T'. This is used to avoid entering into starvation due to the high amount of memory to be used.
- prefixOutFile: this parameter indicates the output file name of this program "prefixOutFile.dict"

We have un-successfully used *dustmask* (from Blast distribution).
Thus, we consider low complexity regions (LCR) must be identified before using this procedure.

dictionary considerations:

1. Accept "ACGT" as valid symbols to conform
2. The program interprets low case symbols as non-valid characters to conform a word, so they are skipped (this is the usual way used by maskers to represent a LCR).
3. Remove non-coding ASCII characters. We have found, in particular the '\r\n' used by MS-DOS coding to represent the new line are not well processed by Linux-like environments (an alternative is to use the dos2unix command)

Output file format:

```
typedef struct {
      //Word compressed in binary format
      word w;
      //Number of ocurrences inside the
      //sequence. This is used to know the
```

```
      //number of locations stored after this
      //struct
      uint64_t num;
} hashentry;

typedef struct {
      //Ocurrence position in the sequence
      uint64_t pos;
      //For multiple sequence files this var
      //reflects in what sequence occurs the
      //word
      uint64_t seq;
} location;
```

4.2. Hits

```
Usage: hits prefixNameX prefixNameY Outfile Ksize
```

Parameters:

- prefixNameX & Y :refers to

  *.dict: index of words-InitPosition-NumberRepetitions; and
- Outfile is in the form of [Diagonal][position in X][sequence in X (for multiple sequence fasta files)][position in Y][sequence in Y (for multiple sequence fasta files)]
- Ksize: prefix matching size (in this way, the dictionary is be comnputed only once for 32-mers but the matches can be computed based on word prefixes)

Output file format:

```
typedef struct {
      //Diagonal where the hit is located
      //This value is calculated as:
      //posX - posY
      int64_t diag;

      //Ocurrence position in sequence X
      uint64_t posX;

      //Ocurrence position in sequence Y
      uint64_t posY;

      //For multiple sequence files this var
      //reflects in what sequence of X file
```

```
        //occurs the word
        uint64_t seqX;

        //For multiple sequence files this var
        //reflects in what sequence of Y file
        //occurs the word
        uint64_t seqY;
} hit;
```

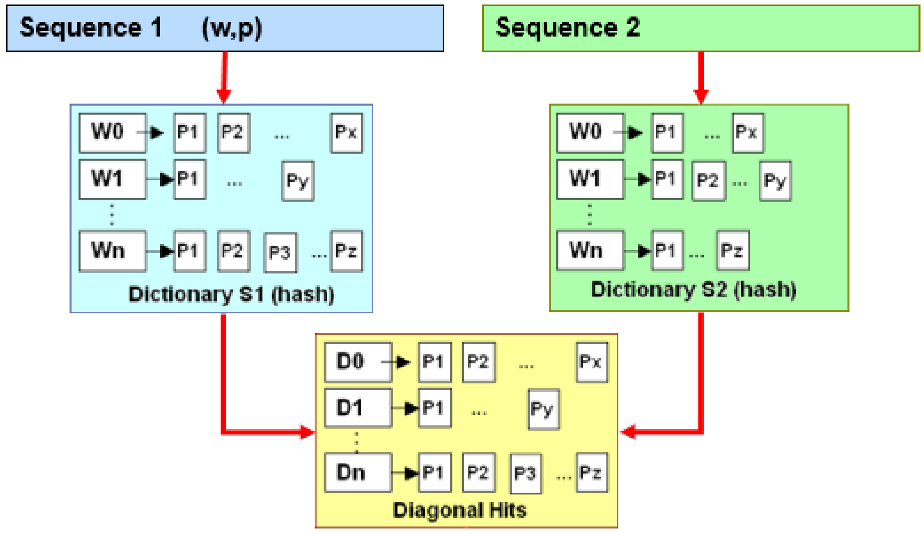

4.3. SortHits

```
Usage: sortHits bufferSize nThreads inputFile Outfile
```

Parameters:

- bufferSize indicates the number of hits to be stored in memory in order to be sorted. Bigger numbers of this parameter give more performance. Recommended value: 10000000
- nThreads indicates the number of parallel POSIX threads that will sort the hits input file. Our experience executing the program tells us that a good value is 32, because in the meanwhile some threads are doing I/O the others are sorting.
- Outfile is ordered first by [Diagonal] and then by [position in X].

4.4. FilterHits

Two hits will be grouped if they occur at a given distance (less than the K-mer size parameter). In this way we reduce the number of hits to be extended. It is also possible to remove all "isolated" hits.

```
Usage: filterHits fileIn fileOut KmerSize filterIsolatedHits
```

Parameters:

- fileIn refers to the hits file coming from the sortHits procedure. This file is sorted first by diagonal and then by position in the sequence X.
- fileOut indicates the output file of this program
- KmerSize indicates the length of the matches in order to know when one hit is starting at a position already covered by a previous hit.
- filterIsolatedHits indicates if a hit that is isolated in one of the diagonal is filtered or not. Usually when two sequences are similar it is normal that in every diagonal they have more than one hit.

Note: here there is a possibility to include in the output how many hits conform each Big-Hit and then compute fragments using only Big-Hits formed by "at least" n simple hits. However, during experiments we verify that for K=32, working with individual hits is fast enough. The main reason for this behaviour is that when hits are ordered, the fragments (next) program is able to collect hits on the same diagonal and then jump all the hits that were incorporated into a given fragment.

The Double-Hit approach used by Blast has demonstrated very powerful to compare short sequences such as genes and proteins. However when working with genomic sequences it is expected large strings of identical fragments (not only by the longer sequences under analysis but other genomic characteristics such as operons, suggest this possibility).

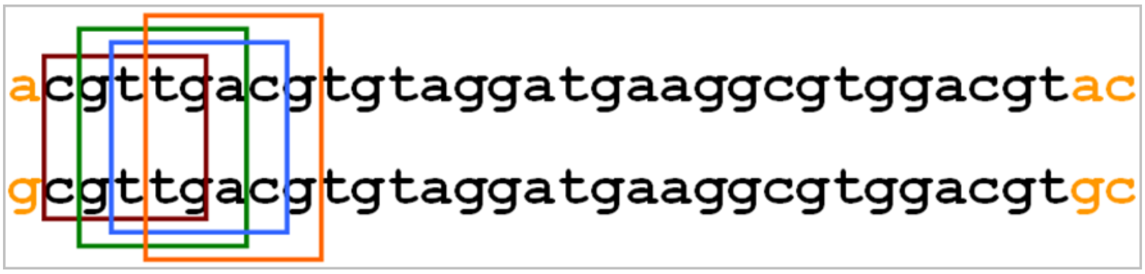

Overlapped hits points for K = 5

Although in the previous slide we have observed that the fragment detection program will avoid consecutive hits to be extended, there are some situations (such parallel implementation) in which is better to fusion the consecutive hits to avoid spurious or duplicated results.

Let's analyze this fact. For fragments with a large identical section several overlapping words will be found (see illustration). All these hits are considered as potential seeds or starting point of fragments. Next step is to extend the seed to identify the real fragments. Under this situation (a) or several seeds will be extended to produce the same fragment; or (b) a clever strategy will discard a-posteriori the consecutive seed once the first one integrate all the seed in one fragment. Potentially, in a parallel implementation, this fact will produce a false estimation of the computational load to be distributed to the different processors, and seeds assigned to different processors will be no identified as close hits.

We have analysed the possibility to extend the seed by using the 2-words matches imported from Blast; or enhancing the seed by let the signal to detect their own boundaries that could be named k-words self-bounded matches.

As it was previously mentioned, the 2-words hits used with high success in program such Blast and FASTA have this good behaviour due to the length of the sequences under analysis (genes or proteins). But in the case of genomic sequences, the probability of consecutive words increase so much and distort the estimation of the computational load. There are more seed than needed since several of this seed are formed by overlapped words.

In the table and the corresponding graph, the number of 1-word hits is displayed together with the number of 2-words blast hits used as seed in the well known application. The self-bounded (N-hits) approach get a notorious reduction of the number of seeds.

Word size	8	10	12
1-Hit	9116866	1053657	419068
Blast	9114584	893780	96933
N-hit	6314262	492683	44458

Here a (numbers) table with the reduction of the number of seed using both approaches

IMPORTANT: since the 1-word matches are ordered by diagonal, the typical array (of length N+M-1) for identification of 2-words matches is no more necessary.

Thus **NOT memory boundaries**!!!

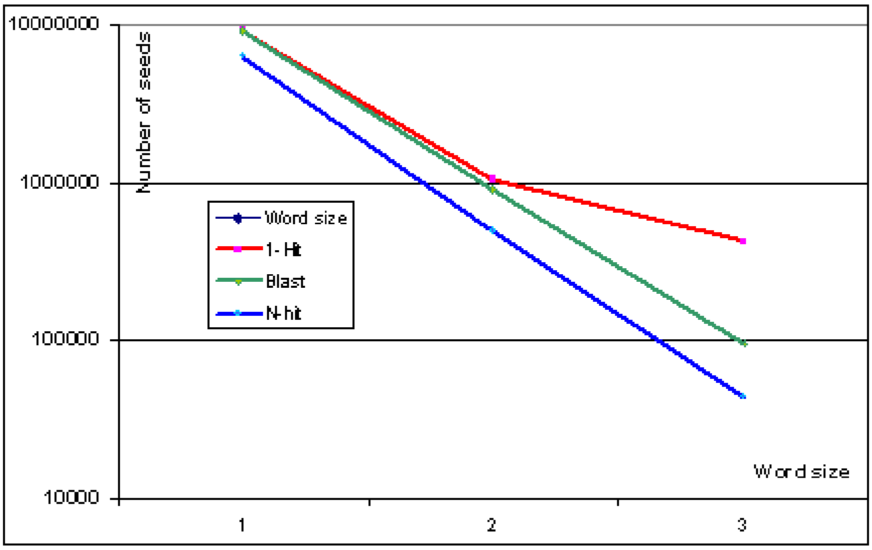

Number of seeds using the 1-word, 2-words-blast and n-hits (log scale).

Here, the **accuracy** of results is evaluated.
The full set of local alignments between two *E.coli* genomes was computed (L1 & L2 ≈ 5Mpb).
The full search space is L1xL2, which is reduced to linear space by identifying 1-word hits (L1+L2 search space) and performing 1.035.923 fragment extensions.

Using a word of length 12; a small identical fragment of length 15 will potentially produce 3x2x1 2-hits seeds which represent the factorial of the difference between the fragment length and the word size .. which is stratospheric for words as large as 30.

However, the strategy of self-bounded seeds (Big-Hits) reduces the search space to 30%.

More important, a 99,98% of the final real fragments are identified (99,99% counting fragments with more than 20 residues length).

Length	number of 1-word hits	n-Hits seeds	Real fragments	Diff.
12	1035923	722265	722283	18
13		218030	218058	28
14		65993	66011	18
15		20164	20183	19
16		6193	6209	16
17		1923	1937	14
18		605	619	14
19		237	239	2
20		75	75	0
21		58	58	0
22		22	22	0
23		22	22	0
24		17	31	14
25		31	44	13
26		14	14	0
27		14		-14
28		15	14	-1
29			14	14
30		18	14	-4
32		14		-14
33		15	15	0
34		2	2	0
25			14	14
36		2		-2
39		14		-14
44		14		-14
46		14	14	0
47		14		-14
48		14		-14
49		14	14	0
63		14	14	0
2031		1	1	0
3150		1	1	0
**TOTAL**	**1035923**	**313564**	**313639**	**75**
		**30,27%**	**99,98%**	

Table of common words of length 12 for the two *E.coli* genomes used in the study. This represent the number of seed point that would be processed to identify fragments. A self-bounded strategy to identify longer seeds by overlapping of single hits allow a reduction to 30% of the seeds needed to examine.The extension of seed to identify fragments (local alignments) perfectly reproduce the results produced by exhaustive strategies.

4.5. FragHits

A fragment is a sub-string present in both sequences whose quality (score) cannot be increased by extending the string in any of both directions. Quality is measured using a scoring scheme. Currently this scoring scheme is hard-coded (4 for identical symbols, -1 for N-N matches and -4 for different symbols) but we have also available a version which uses a user-specified scoring matrix. The program also accounts the number of identical symbols in the fragment.

It is easy to deduce that a fragment starts and ends with an identical symbol and eventually grows until reach a maximum quality or the sequences ends.

To avoid poorly scored fragments, a filtering strategy is used, based on similarity or statistical significance. Thus, when the borders of the fragment are detected, the maximum score is computed in a level of similarity is obtained. In other cases, the p-value is computed for the corresponding fragment.

Noteworthy to observe, the fragment with higher score is not necessarily the best fragment from the similarity point of view, being possible –and really frequent- that a discarded long fragment could contains one or more shorter but well-conserved sub-fragments that satisfy filtering requirements.

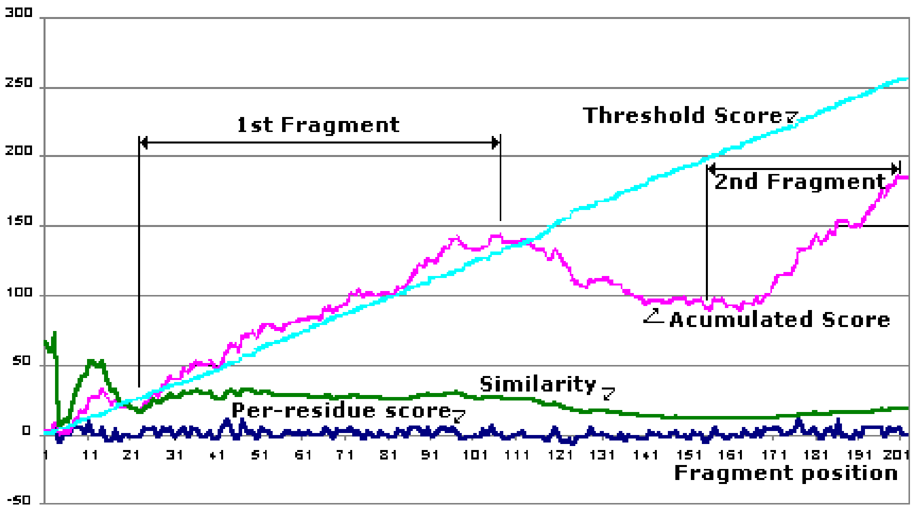

Typical fragment detection algorithms that apply quality assessment at the end of fragment detection. In the picture (bottom part) the per-residue score and the similarity level are shown. The growing line represents the accumulated score that should be needed at each point by the fragment to be accepted as an interesting fragment (over the similarity threshold). The hill-shaped line correspond to the real score the fragment

obtain at each point. This fragment should be discarded if a similarity threshold is used, because at the end of the fragment although the score is maximum, the similarity does not. Computing at each point the criterion that will be used to filter the fragment should produce two shorter but well-conserved fragments. (a length based threshold score can also be used). Although a slightly increase in CPU-time is needed, better results are obtained.

The following figure illustrates an example of how the actual HSP score is calculated:

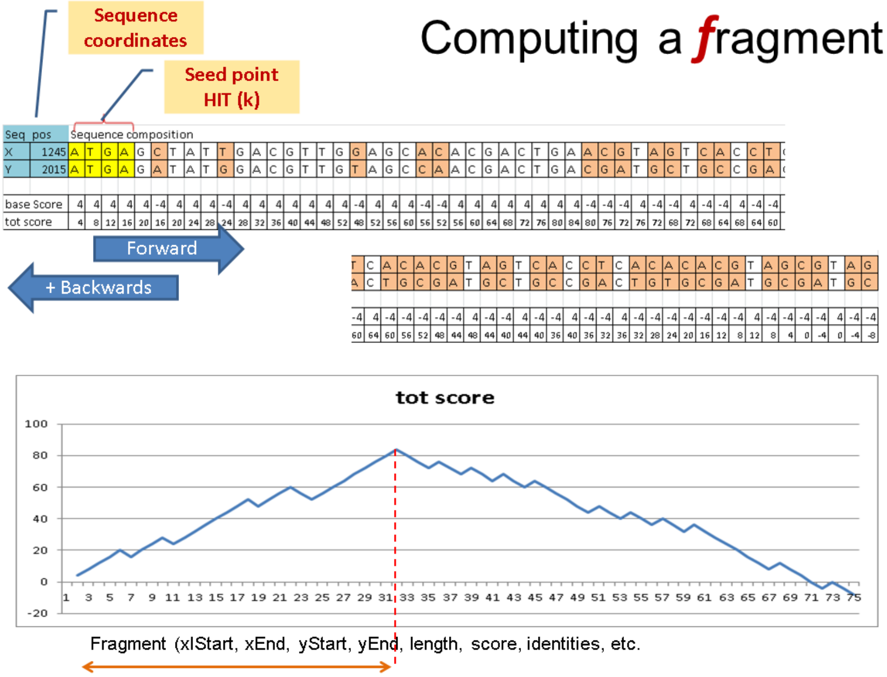

In this case, hits are used as seed points to extend the fragment. A backward step is also included to extend the fragment in the opposite direction from the hit.

```
Usage: FragHits SeqX.file SeqY.file HitsFile Out.file Lmin
SimThr WordSize fixedLen strand
```

Parameters:

- SeqX.file and SeqY.file are the sequences in FASTA format.
- HitsFile (binary, ordered and filtered).
- Out file (binary) will save a fragment with the format:

```
struct FragFile {
    //Diagonal where the frag is located
    //This value is calculated as:
```

```
    //posX - posY
    int64_t diag;

    //Start position in sequence X
    uint64_t xStart;

    //Start position in Sequence Y
    uint64_t yStart;

    //End position in Sequence X
    uint64_t xEnd;

    //End position in Sequence Y
    uint64_t yEnd;

    //Fragment Length
    //For ungaped aligment is:
    //xEnd-xStart+1
    uint64_t length;

    //Number of identities in the
    //fragment
    uint64_t ident;

    //Score of the fragment. This
    //depends on the score matrix
    //used
    uint64_t score;

    //Percentage of similarity. This
    //is calculated as score/scoreMax
    //Where score max is the maximum
    //score possible
    float similarity;

    //sequence number in the 'X' file
    uint64_t seqX;

    //sequence number in the 'Y' file
    uint64_t seqY;

    //synteny block id
    int64_t block;

    //'f' for the forward strain and 'r' for the
reverse
```

```
    char strand;
};
```

- Lmin: minimal fragment length.
- SimThr: Similarity Threshold (to obtain all fragments use a low value, or zero).
- WordSize indicates the K value used to calculate the hits (seed points) based on the computed sequence dictionaries.
- FixedLen indicates whether the length parameter should be taken into account as the actual value or as the percentage of the length of the input sequences. This parameter has more sense when comparing multiple sequence fasta files. Allowed values: 1 (actual value), 0 (to be considered as percentage).
- Strand indicates whether we are computing the forward or the reverse strand fragments. Allowed values: f (forward), r (reverse).

4.6. Additional programs

readDict

This program will read the calculated dictionary and will produce the following output:

```
<word>: num:<number of ocurrences> : <list of occurrences>
```

```
Usage: readDict sequence.Dict
```

Parameters:

- Sequence.dict refers to the dictionary computed for a given sequence.

Example:

```
../readDict sequence.dict
AAAAAAAAAAAAAAAAAAAAAAAAAAAAAAAA  : num=7      :0 16 32 48
64 80 96
AAAAAAAAAAAAAAAACCCCCCCCCCCCCCCC  : num=7      :1 17 33 49
65 81 97
AAAAAAAAAAAAGGGGGGGGGGGGGGGGGGGG  : num=7      :2 18 34 50
66 82 98
AAAAAAATTTTTTTTTTTTTTTTTTTTTTTTT  : num=7      :3 19 35 51
67 83 99
CCCCCCCCCCCCCCCCCAAAAAAAAAAAAAAA  : num=6      :4 20 36 52
68 84
CCCCCCCCCCCCCCCCCCCCCCCCCCCCCCCC  : num=6      :5 21 37 53
69 85
CCCCCCCCCCCCCCCGGGGGGGGGGGGGGGGG  : num=6      :6 22 38 54
70 86
```

```
CCCCCCCCCCCCTTTTTTTTTTTTTTTTTTTT  : num=6      :7 23 39 55
71 87
GGGGGGGGGGGGGCCCCCCCCCCCCCCCCCCC  : num=6      :9 25 41 57
73 89
GGGGGGGGGGGGGGGGAAAAAAAAAAAAAAAA  : num=6      :8 24 40 56
72 88
GGGGGGGGGGGGGGGGGGGGGGGGGGGGGGGG   :  num=6       :10  26  42
58 74 90
GGGGGGGGTTTTTTTTTTTTTTTTTTTTTTTT   :  num=6       :11  27  43
59 75 91
TTTTTTTTTTTTTAAAAAAAAAAAAAAAAAAA   :  num=6       :12  28  44
60 76 92
TTTTTTTTTTTTTCCCCCCCCCCCCCCCCCCC   :  num=6       :13  29  45
61 77 93
TTTTTTTTTTTTTTGGGGGGGGGGGGGGGGGG   :  num=6       :14  30  46
62 78 94
TTTTTTTTTTTTTTTTTTTTTTTTTTTTTTTT   :  num=6       :15  31  47
63 79 95
Num.Words=16 MaxFreq=7  TotRepeat=100 AveragFreq=6.250000
```

alignFrags

Initially designed as debug program to check the fragments detected, but it can also be used to produce all the alignments, or the program can be easily modified to produce the list of fragment coordinates

```
Usage: alignFrags SeqX.fa SeqY.fa frags.file
```

Parameters:

- SeqX.fa refers to the reference input sequence used to calculate the fragments.
- SeqY.fa refers to the query input sequence used to calculate the fragments.
- Frags.file refers to the computed fragments shared between sequence X and sequence Y.

Output example:

```
X=45491783  Y=158660077  L=159  Matches=129   -------
GCTCTCCTCGCCCTCCAGCAGCTTCCTGTAGGTGGCGATCTCTACGTCCAGGGCCAGCTT
||||||||||||||||||||||||||||||||||||||||||   ||| || ||||||||
GCTCTCCTCGCCCTCCAGCAGCTTCCTGTAGGTGGCGATCTCGGTGTCAAGCGCCAGCTT
GACATTCATCAGCTCCTGGTATTCACGCAGAGGCCGCGCCATGTCCTGCTTGGCCCGCTG
||| ||||||||||||||||| || | |||  ||| ||||||  |  |||||||||  ||
GACGTTCATCAGCTCCTGGTACTCTCACAGCTGCCTCGCCATAGCTGGCTTGGCCCTTTG
CAGAACGGCCTCCAGCTCGGACAACTTGGCCTTGGCATC
| |  |   ||||| ||| | || |||||| |||| |||
```

```
CGGGGCATTCTCCATCTCAGTCAGCTTGGCATTGGTATC
------------------------------------------------Matches=129
```

5. Results quality

Although the performance aspects of GECKO's design are crucial, the production of high quality results is equally important. In this section we explain how we evaluated the quality of the results produced by our algorithm versus the other applications using the same parameters. The rationale behind our evaluation was to compare the coverage of the HSPs detected by each algorithm. To avoid biases in the evaluation we decide to obtain a consensus set of reference HSPs. This set is composed of those HSPs reported by at least half of the reference algorithms. The HSPs produced by GECKO were then mapped over the reference HSPs and the percentage of coverage recorded as a measurement of result quality. This means that matching positions reported by the consensus HSP reference and not reported by GECKO will push down the quality and vice versa. There are more sophisticated ways of comparing the results, such as only considering coding regions, or by qualifying and weighting matches depending on sequence type or section. However, we decided not to use these methods as they can incorporate noise or biases into the evaluation.

We have performed different experiments for closely and remotely related sequences in order to thoroughly study the results quality.

5.1. Closely related sequences

Following the previously described procedure with the results of the pairwise test described in the paper, the evaluation determined that in our experiments GECKO detected 3% more HSPs than the consensus set. Moreover, GECKO obtained a larger dataset while maintaining identity values over 65%, thus representing the identification of additional statistically-significant HSPs.

In the following sections we will summarize some examples of the performed experiments showing the HSPs we report and the other don't and viceversa. We will show also the alignment of those we are not taking into account to point out they are not good alignments. Additionally, we will also provide an example

TLCV vs. TYLCV-lr2

As explained before the result of the comparison based on coverage is the following:

Gecko cov.	87,45%
Consensus cov.	83,17%
Diference	4,28%

In this plot we can see that the HSPs that are contributing to GECKO's coverage are concentrated in the zone of 70 residues and also in short segments. Given the fact of the short sequence length, and also that the HSPs are non-overlapping, we believe the previously mentioned segments are the ones contributing to the better coverage.

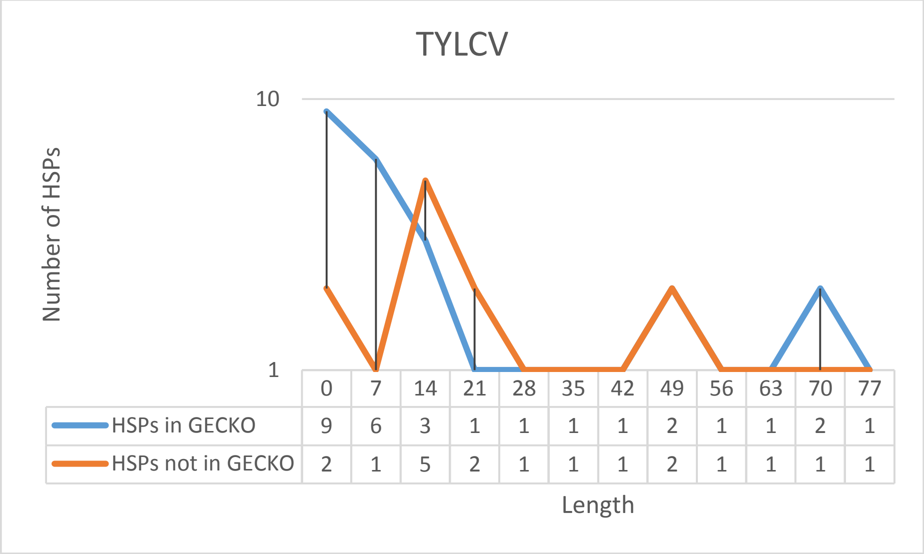

Buchnera APS vs. Buchnera 5A

As explained before the result of the comparison based on coverage is the following:

**Gecko cov.**	**100,00%**
**Consensus cov.**	**99,99%**
**Difference**	**0,01%**

In this comparison the difference in coverage is minimal so the plot of the HSPs present in GECKO and not in the others is not so significant since the lengths in the X axis are very short.

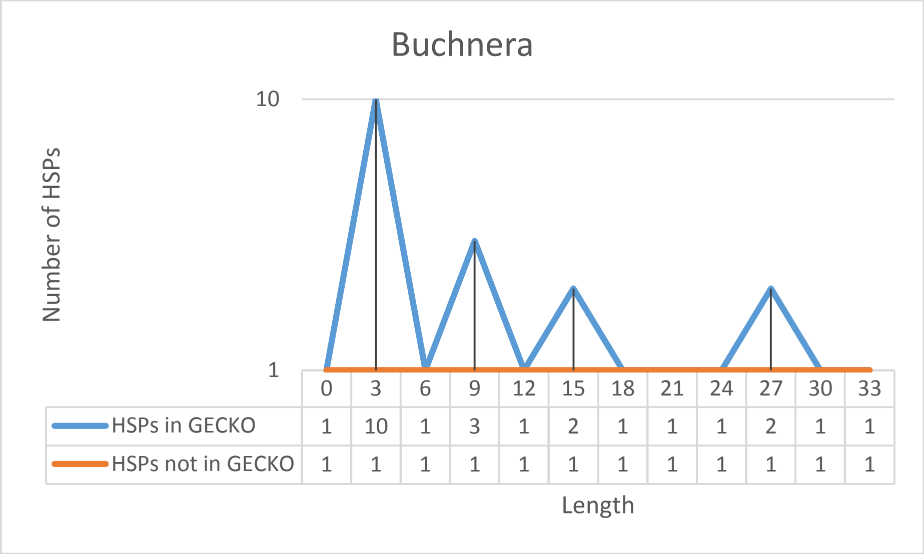

E.colik12 vs. E.coliO157

As explained before the result of the comparison based on coverage is the following:

**Gecko cov.**	**90,19%**
**Consensus cov.**	**88,93%**
**Difference**	**1,26%**

The frequencies of the HSPs of different lengths found in one party and not in the other can be visualized in the next figure (Y axis in logarithmic scale).

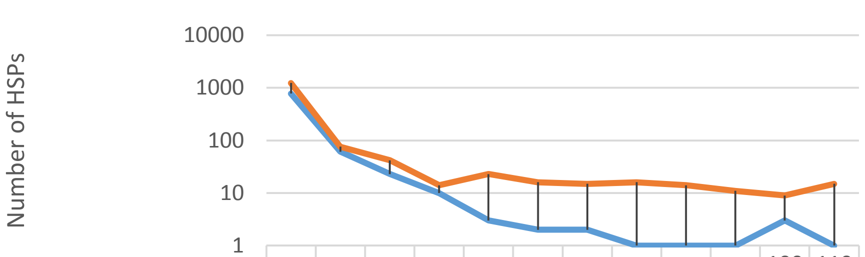

	0	100	200	300	400	500	600	700	800	900	1000	1100
HSPs not in GECKO	775	61	23	10	3	2	2	1	1	1	3	1
HSPs in GECKO	1235	76	42	14	23	16	15	16	14	11	9	15

One example of the long HSPs reported by GECKO and not the others is the following:

```
X=3184117  Y=3911085  L=1332  Matches=1325
TAGACTGGCCCCCTGAATCTCCAGACAACCAATATCACTTAAATAAGTGATAGTCTTAAT
||||||||||||||||||||||||||||||| ||||||||||||||||||||||||||||
TAGACTGGCCCCCTGAATCTCCAGACAACCAGTATCACTTAAATAAGTGATAGTCTTAAT

ACTAGTTTTTAGACTAGTCATTGGAGAACAGATGATTGATGTCTTAGGGCCGGAGAAACG
||||||||||||||||||||||||||||||||||||||||||||||||||||||||||||
ACTAGTTTTTAGACTAGTCATTGGAGAACAGATGATTGATGTCTTAGGGCCGGAGAAACG

CAGACGGCGTACCACACAGGAAAAGATCGCAATTGTTCAGCAGAGCTTTGAACCGGGGAT
||||||||||||||||||||||||||||||||||||||||||||||||||||||||||||
CAGACGGCGTACCACACAGGAAAAGATCGCAATTGTTCAGCAGAGCTTTGAACCGGGGAT

GACGGTCTCCCTCGTTGCCCGGCAACATGGTGTAGCAGCCAGCCAGTTATTTCTCTGGCG
||||||||||||||||||||||||||||||||||||||||||||||||||||||||||||
GACGGTCTCCCTCGTTGCCCGGCAACATGGTGTAGCAGCCAGCCAGTTATTTCTCTGGCG

TAAGCAATACCAGGAAGGAAGTCTTACTGCTGTCGCCGCCGGAGAACAGGTTGTTCCTGC
|||||||||| |||||||||||||||||||||||||||||||||||||||||||||||||
TAAGCAATACTAGGAAGGAAGTCTTACTGCTGTCGCCGCCGGAGAACAGGTTGTTCCTGC

CTCTGAACTTGCTGCCGCCATGAAGCAGATTAAAGAACTCCAGCGCCTGCTCGGCAAGAA
||||||||||||||||||||||||||||||||||||||||||||||||||||||||||||
CTCTGAACTTGCTGCCGCCATGAAGCAGATTAAAGAACTCCAGCGCCTGCTCGGCAAGAA

AACGATGGAAAATGAACTCCTCAAAGAAGCCGTTGAATATGGACGGGCAAAAAAGTGGAT
||||||||||||||||||||||||||||||||||||||||||||||||||||||||||||
AACGATGGAAAATGAACTCCTCAAAGAAGCCGTTGAATATGGACGGGCAAAAAAGTGGAT
```

```
AGCGCACGCGCCCTTATTGCCCGGGGATGGGGAGTAAGCTTAGTCAGCCGTTGTCTCCGG
||||||||||||||||||||||||||||||||||||||||||||||||||||||||||||
AGCGCACGCGCCCTTATTGCCCGGGGATGGGGAGTAAGCTTAGTCAGCCGTTGTCTCCGG

GTGTCGCGTGCGCAGTTGCACGTCATTCTCAGACGAACCGATGACTGGATGGATGGCCGC
|||||||||||||||||| |||||||||||||||||||||||||||||||||||||||||
GTGTCGCGTGCGCAGTTGTACGTCATTCTCAGACGAACCGATGACTGGATGGATGGCCGC

CGCAGTCGTCACACTGATGATACGGATGTGCTTCTCCGTATACACCATGTTATCGGAGAG
||||||||||||||||||||||||||||||||||||||||||||||||||||||||||||
CGCAGTCGTCACACTGATGATACGGATGTGCTTCTCCGTATACACCATGTTATCGGAGAG

CTGCCAACGTATGGTTATCGTCGGGTATGGGCGCTGCTTCGCAGACAGGCAGAACTTGAT
||||| ||||||||||||||||||||||||||||||||||||||||||||||||||||||
CTGCCCACGTATGGTTATCGTCGGGTATGGGCGCTGCTTCGCAGACAGGCAGAACTTGAT

GGTATGCCTGCGATCAATGCCAAACGTGTTTACCGGATCATGCGCCAGAATGCGCTGTTG
||||||||||||||||||||||||||||||||||||||||||||||||||||||||||||
GGTATGCCTGCGATCAATGCCAAACGTGTTTACCGGATCATGCGCCAGAATGCGCTGTTG

CTTGAGCGAAAACCTGCTGTACCGCCATCGAAACGGGCACATACAGGCAGAGTGGCCGTG
||||||||||||||| ||||||||||||||||||||||||||||||||||||||||||||
CTTGAGCGAAAACCTACTGTACCGCCATCGAAACGGGCACATACAGGCAGAGTGGCCGTG

AAAGAAAGCAATCAGCGATGGTGCTCTGACGGGTTCGAGTTCTGCTGTGATAACGGAGAG
||||||||||||||||||||||||||||||||||||||||||||||||||||||||||||
AAAGAAAGCAATCAGCGATGGTGCTCTGACGGGTTCGAGTTCTGCTGTGATAACGGAGAG

AGACTGCGTGTCACGTTCGCGCTGGACTGCTGTGATCGTGAGGCACTGCACTGGGCGGTC
||||||||||||||||||||||||||||||||||||||||||||||||||||||||||| 
AGACTGCGTGTCACGTTCGCGCTGGACTGCTGTGATCGTGAGGCACTGCACTGGGCGGTG

ACTACCGGCGGCTTCAACAGTGAAACAGTACAGGACGTCATGCTGGGAGCGGTGGAACGC
||||||||||||||||||||||||||||||||||||||||||||||||||||||||||||
ACTACCGGCGGCTTCAACAGTGAAACAGTACAGGACGTCATGCTGGGAGCGGTGGAACGC

CGCTTCGGCAACGATCTTCCGTCGTCTCCAGTGGAGTGGCTGACGGATAATGGTTCATGC
||||||||||||||||||||||||||||||||||||||||||||||||||||||||||||
CGCTTCGGCAACGATCTTCCGTCGTCTCCAGTGGAGTGGCTGACGGATAATGGTTCATGC

TACCGGGCTAATGAAACACGCCAGTTCGCCCGGATGTTGGGACTTGAACCGAAGAACACG
||||||||||||||||||||||||||||||||||||||||||||||||||||||||||||
TACCGGGCTAATGAAACACGCCAGTTCGCCCGGATGTTGGGACTTGAACCGAAGAACACG

GCGGTGCGGAGTCCGGAGAGTAACGGAATAGCAGAGAGCTTCGTGAAAACGATAAAGCGT
||||||||||||||||||||||||||||||||||||| ||||||||||||||||||||||
GCGGTGCGGAGTCCGGAGAGTAACGGAATAGCAGAGAACTTCGTGAAAACGATAAAGCGT

GACTACATCAGTATCATGCCCAAACCAGACGGGTTAACGGCAGCAAAGAACCTTGCAGAG
||||||||||||||||||||||||||||||||||||||||||||||||||||||||||||
GACTACATCAGTATCATGCCCAAACCAGACGGGTTAACGGCAGCAAAGAACCTTGCAGAG
```

```
GCGTTCGAGCATTATAACGAATGGCATCCGCATAGTGCGCTGGGTTATCGCTCGCCACGG
||||||||||||||||||||||||||||||||||||||||||||||||||||||||||||
GCGTTCGAGCATTATAACGAATGGCATCCGCATAGTGCGCTGGGTTATCGCTCGCCACGG

GAATATCTGCGGCAGCGGGCTTGTAATGGGTTAAGTGATAACAGATGTCTGGAAATATAG
||||||||||||||||||||||||||||||||||||||||||||||||||||||||||||
GAATATCTGCGGCAGCGGGCTTGTAATGGGTTAAGTGATAACAGATGTCTGGAAATATAG

GGGCAAATCCAG
||||||||||||
GGGCAAATCCAG
```

As you can see the alignment is pretty good, in fact the identity level calculated as the matches divided by the length is of 99,47%

D. Melanogaster chr2R vs. D. Pseudoobscura chr3

**Gecko cov.**	**90,19%**
**Consensus cov.**	**88,93%**
**Diference**	**1,26%**

In this comparison with larger sequences the state of the art software is joining small HSPs to conform long alignments. This is the explanation of why GECKO is reporting more HSPs only in the first range of the plot and the rest software has longer HSPs.

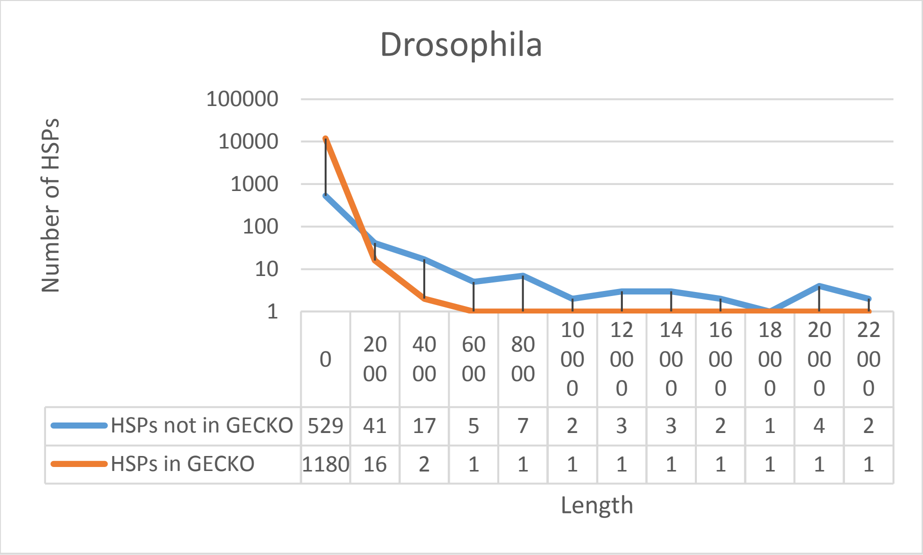

One example of the long HSPs they are producing that is not present in our software is:

```
X=15254265  Y=7423834  L=888  Matches=569
AGAGACTCTGCTCGTTGGATCTACAGCTCGCCACCTGTGATTCCAATGAACATAAACCTT
|| ||||||||||| |||||||| ||| |||| ||   | | || || |||||  | ||
AGGGACTCTGCTCGGTGGATCTATAGCACGCCTCCACAGCTCCCCATAAACATCGATCTG

ACCCAGCTGTCTAAACAATCTCTGGATGGCAACAAGGTACGATATGAGTACAACCTGAGG
||  |  | |||||   ||||||||     |||||||| || || |||| || |||| ||
ACGGAAGTCTCTAAGGCATCTCTGGGACAGAACAAGGTTCGCTACGAGTTCAGCCTGCGG

GCCAGCGATCGCGTTATGATGTACATAGATCCGCTTGACAATGTCAAGGTGACAGATTGG
|||||||||||||| ||||| ||||||| |||  | ||  | ||||||||  | || |||
GCCAGCGATCGCGTCATGATCTACATAGCTCCAATGGATCAGGTCAAGGTCTCGGACTGG

TCCTTCGATCATACACCTTTGGTGGAGAAGCACACACCTCCCTATCTGATCTATGCCATA
|| || ||  | |||||  |||  |||  |||||| || |||||||| || ||   ||||
TCATTTGACAAGACACCACTGGATGAGGGGCACACTCCGCCCTATCTTATGTACCACATA

TACTCCCAAACCGAGGAGCCGCTCAACTTTTGGGTGGAGCTCGAGCACGAGGAGGGCAAC
|| ||    |||||||||||  || |||| ||||||||||||||||||||||   | ||
TATTCTATGACCGAGGAGCCCTTCGACTTCTGGGTGGAGCTCGAGCACGAGGTCAGTAAT

ACAGACGGACCCTACATGAAGCTGGTCGTATCCGAGCACTTCCAATACCATCCAGAGTAC
|| || ||||| ||  | || ||||| || || ||||||||||  |||||    ||| ||
ACGGAGGGACCGTATTTTAAACTGGTAGTCTCGGAGCACTTCCTCTACCACAAGGAGCAC

TACACCGAGGAATACAAGGAATTCCTCGCAACCTTCCCCGATTGGACATATACCACCGAT
||||||||||| | |  ||| |||||||  || |||||||| ||||| || |||||||||
TACACCGAGGACTTCCGGGAGTTCCTCGGCACTTTCCCCGACTGGACCTACACCACCGAT

TGGTTCTCGGCCCTCGAGAGCTGGATTGTTTAGGCGGCGATGAGAATATAGACTCCAAAG
||||| ||| || | || |||||| |  | || || ||||  ||  |  ||  | ||  |
TGGTTTTCGTCCTTGGAAAGCTGGGTATTGTAAGCAGCGAATAGTTTTAAGGATACATCG

TTGGCGTATCTTAATAAATAATTTATAGTATATTTTAATAAAATTACAAAGTAGTAACTA
 |    || ||||||| | ||||||||||||||||||||||  | |  |     | |
ATTTGTTAACTTAATACAAAATTTATAGTATATTTTAATAAGCTAATTATAATCTTAAAT

CAGTGTTCTAATCGTAAATACTAATACAATTTAGGACGTGCCATGCTTATCGACACTTTG
|      |   |   |  |    |   || |       ||        ||   ||   |
CTTAAAGCATTTATGACGTGACCAGGAAACTGGCACTTTGTTCGTTGGATTACCAGGATA

TTCGTTGGATTAGGTAAAATTGGTACACTCCATCAACAGTACCAGAATTAAGAGTAGCTG
        |            || |||  |    |  |  |  |     |        ||
ACGAGGATAAGGCTATCCCGGGGGACATACATGGAGAAACATTAACCAAATTTAAGAGTG

AGGTGTCATGGCAAAAATGGGATTGAGTTGAGTTCGTGAAGGAACTTTGAGTCTACACTT
|            |    |     |  | |      |   |    |    |   ||||||
ACTGAATGGCAACATTGGGATTCAGTTTGGTTCGATTATCGACTGTACTGGGGAACACTT

AAAGCTGCCAACTATTGTAGGATCCCAGCCAGGAGCTAACATGCGCCCACGGCGGAAACG
| |||||||| || |||||||| || | ||||||||| ||||||||||||||||||||||
```

```
AGAGCTGCCAGCTGTTGTAGGAACCAATCCAGGAGCTTACATGCGCCCACGGCGGAAACG

TGGCCAGGAACTCACGGAAATCCTCCGTAATATACATGTCATCGTGTATTCGATGACCCA
||||||||||||| | ||| |||||    || |||||||||| |||| |||  || ||||
TGGCCAGGAACTCCCTGAACTCCTCATCTATGTACATGTCATGGTGTGTTCTGTGTCCCA

TTAGGGCTATTTTGAAAGTGCCTCCCGACCAATTTGGATCTTCGTGCT
||| ||| || ||||| ||| |||| ||||| ||    | ||| ||||
TTATGGCAATCTTGAACGTGTCTCCAGACCAGTTATCGTTTTCATGCT
```

As you can see there are some zones with good matches (the ones we are reporting as shorter segments) but there are also zones with no high identity. In fact the number of matches/identities of the whole segment is of 569 with a length of 888, what gives us an identity value of 64.08%.

5.2. Remotely related sequences

It is important to have results of the same quality for closely related sequences, but is also important to check whether this is also applicable to remotely related sequences. This is the reason why we performed an additional experiment where we compared closely and remotely related sequences. This experiment compares the following sequences:

Species (Strain)	Acc. number
M_Bovis_Hubei-1	NC_015725.1
M_bovis_PG45	NC_014760.1
M_agalactiae	NC_013948.1
M_agalactiae_PG2	NC_009497.1
M_fermentans	NC_014552.1
M_fermentans_M64	NC_014921.1

These sequences have the following relations with each other (this tree representation could not be equal to the phylogenetic tree of mycoplasma):

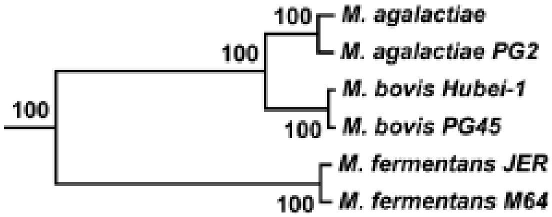

As anybody could expect, the comparisons between nodes placed close in the tree will correspond to closely related sequences comparisons, and distant nodes will correspond with the remotely related sequences comparisons.

Performing an all-vs-all study using GECKO, we obtained the following graphical output:

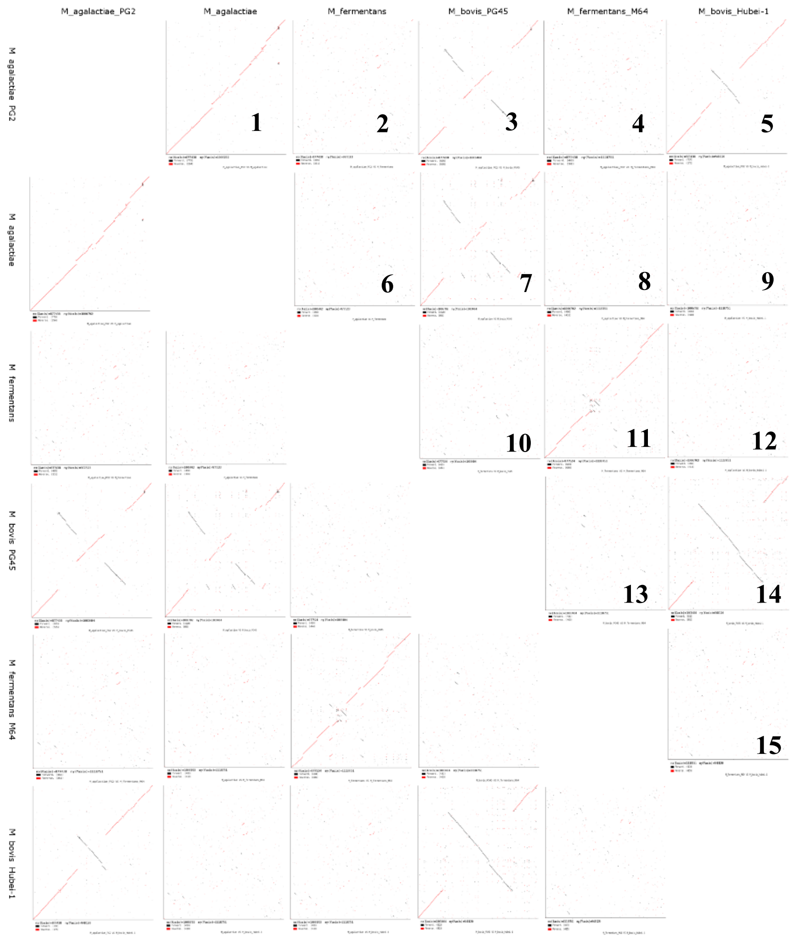

We can observe that there are some subfigures with diagonal stretches, what means that the sequences are closely related and some other with almost no content what means that they are remotely related.

In the following table we show the coverage results as shown in the closely related sequences case of Section 5.1. (Numbers in the first column correspond to the ones shown in the previous figure)

Comparison	Method	Value
1	Gecko cov.	88,51%
	Consensus cov.	86,90%
	Diference	1,62%
2	Gecko cov.	4,66%
	Consensus cov.	0,96%
	Diference	3,70%
3	Gecko cov.	64,82%
	Consensus cov.	13,55%
	Diference	51,27%
4	Gecko cov.	4,99%
	Consensus cov.	0,97%
	Diference	4,02%
5	Gecko cov.	64,65%
	Consensus cov.	50,89%
	Diference	13,76%
6	Gecko cov.	3,88%
	Consensus cov.	2,94%
	Diference	0,94%
7	Gecko cov.	64,90%
	Consensus cov.	48,98%
	Diference	15,92%
8	Gecko cov.	4,01%
	Consensus cov.	2,94%
	Diference	1,07%
9	Gecko cov.	56,50%
	Consensus cov.	40,55%
	Diference	15,95%
10	Gecko cov.	4,25%
	Consensus cov.	2,70%
	Diference	1,56%
11	Gecko cov.	93,97%
	Consensus cov.	93,24%
	Diference	0,73%
12	Gecko cov.	3,58%
	Consensus cov.	2,73%

	Diference	0,85%
13	Gecko cov.	4,01%
	Consensus cov.	2,71%
	Diference	1,30%
14	Gecko cov.	90,19%
	Consensus cov.	79,19%
	Diference	11,00%
15	Gecko cov.	2,92%
	Consensus cov.	0,47%
	Diference	2,44%

The previous table confirms once more that for closely related sequences the results are better, but it also states that the results are equally good for remotely related sequences being comparable with the results obtained with the state of the art methods.

In addition to the previous set of remotely related sequences comparisons, we wanted to analyze it also for longer ones as well. In order to do this, we took the results of the comparisons of chromosome 1 from Homo Sapiens (HS) vs Ratus Norvegicus (RN) and Pan Troglodytes (PT) vs Bos Taurus (BT). The table below contain the coverage values for both comparisons.

Comparison	Method	Value
HS-RN	Gecko cov.	0,03%
	Consensus cov.	0,03%
	Diference	0,00%
PT-BT	Gecko cov.	0,10%
	Consensus cov.	0,03%
	Diference	0,07%

The table confirms once more that the quality is comparable and superior in this different scenario.

5.3. Study of the number of identities

We have evaluated the difference between the coverage obtained by the HSPs of GECKO and the ones of the consensus set. But, one could think that this difference is caused by HSPs with low identity values in the case of GECKO. In order to illustrate that this is far to be the case, we present here a study of the HSPs' identities of GECKO

compared to LASTZ (Gepard, MUMmer and Mauve are not included because they do not report identity values in their standalone output).

The following table contains some statistical information (Average, standard deviation, minimum, maximum and number of HSPs) about the distribution of the identity values of the HSPs reported in some of the comparisons performed in previously shown studies.

	Buchnera		E.coli		Drosophila		HS-RN		PT-BT	
	lastz	gecko	lastz	gecko	lastz	gecko	lastz	gecko	lastz	gecko
avg.	73,83	**82,61**	90,08	**93,97**	**84,62**	81,79	72,33	**83,68**	68,59	**68,98**
std. Dev.	6,78	**3,82**	7,94	**6,63**	7,65	**6,54**	**1,17**	2,97	**0,41**	4,08
min	63,70	**79,07**	55,70	**70,33**	54,90	**69,72**	69,40	**80,04**	**66,50**	65,01
max	**100,00**	**100,00**	**100,00**	**100,00**	**100,00**	**100,00**	74,50	**97,78**	82,50	**96,30**
#HSPs	126	90	11218	1547	40656	13428	881	5527	12529	5565

We highlighted in bold:

1. The higher value of the identity average, because indicates a better set of HSPs (although we know the drawbacks of the average).
2. The lower value of the standard deviation, since the average can makes us think we have a better/worse set when some outliers are present.
3. The higher value of the minimum identity value, because this give us the value of the worse HSP contained in the resulting set.
4. The higher value of the maximum identity value, since it indicated the better HSP reported.

GECKO outperforms LASTZ results in most of the cases except in:

1. The average in the Drosophila comparison, which is a bit lower, but actually this is somehow compensated by the lower standard deviation.
2. The standard deviation in the HS-RN comparison, which is a bit higher but this is because the identity range of the HSPs is wider than the one of LASTZ.
3. The standard deviation in the PT-BT comparison, which is a bit higher but this is again because of GECKO's wider identity range.
4. The minimum identity value in the PT-BT comparison, which is a bit lower but comparable to the one obtained by LASTZ.

In order to give more details about the distribution of HSPs according to their identity values, we present below some normalized graphs (eliminating the dependency of the total number of reported HSPS) that could help to understand the better values obtained by GECKO. The X-axis indicate the identity range and the Y-axis the percentage of the total number of HSPs actually contained in each range. The X-axis starts at 60 just because there are no HSPs below the 50% of identity.

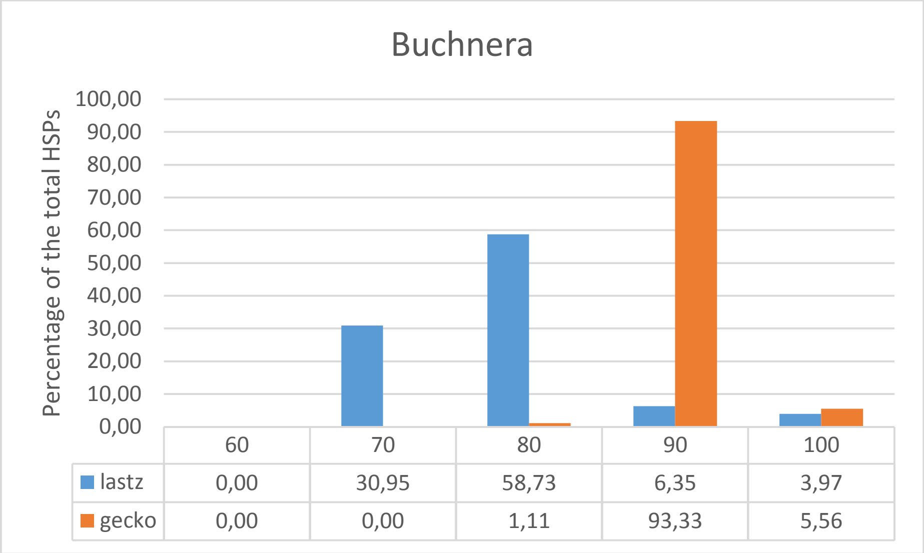
Buchnera
Percentage of the total HSPs
100,00
90,00
80,00
70,00
60,00
50,00
40,00
30,00
20,00
10,00
0,00
60
70
80
90
100
lastz
0,00
30,95
58,73
6,35
3,97
gecko
0,00
0,00
1,11
93,33
5,56

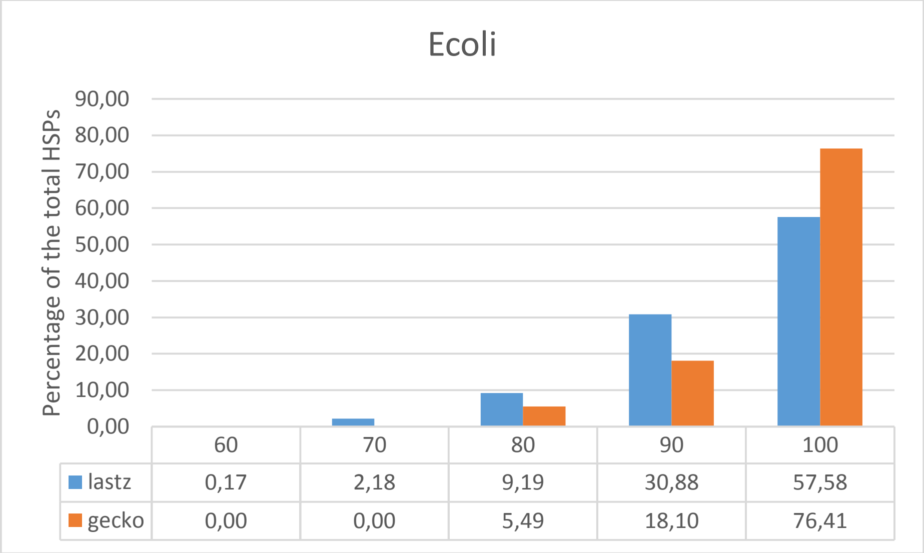
Ecoli
Percentage of the total HSPs
90,00
80,00
70,00
60,00
50,00
40,00
30,00
20,00
10,00
0,00
60
70
80
90
100
lastz
0,17
2,18
9,19
30,88
57,58
gecko
0,00
0,00
5,49
18,10
76,41

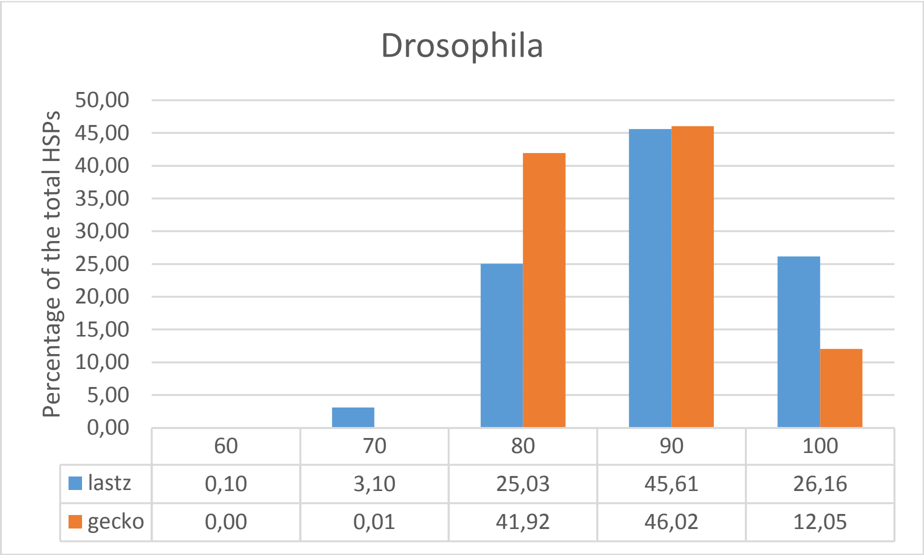
Drosophila
Percentage of the total HSPs
50,00
45,00
40,00
35,00
30,00
25,00
20,00
15,00
10,00
5,00
0,00
60 70 80 90 100
lastz 0,10 3,10 25,03 45,61 26,16
gecko 0,00 0,01 41,92 46,02 12,05

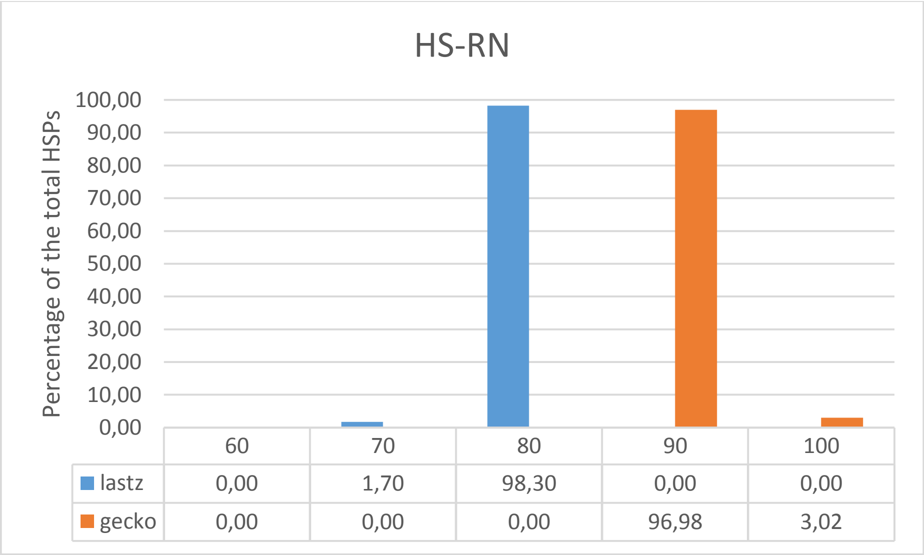
HS-RN
Percentage of the total HSPs
100,00
90,00
80,00
70,00
60,00
50,00
40,00
30,00
20,00
10,00
0,00
60 70 80 90 100
lastz 0,00 1,70 98,30 0,00 0,00
gecko 0,00 0,00 0,00 96,98 3,02

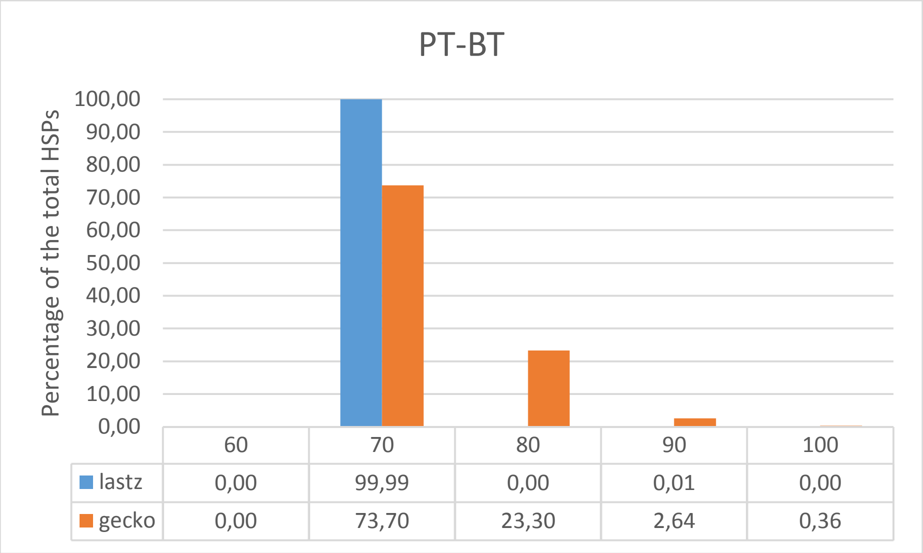

	60	70	80	90	100
lastz	0,00	99,99	0,00	0,01	0,00
gecko	0,00	73,70	23,30	2,64	0,36

After analyzing the results both in terms of coverage and identity, we can say that our results are superior in both situations for the presented studies. This makes us believe it will behave similarly in other comparisons.

5.4. Alignathon dataset study

First, we would like to stress that GECKO was designed to calculate High-Scoring Segment Pairs (HSPs) which, by definition, do not contain gaps, in contrast to the gapped alignments contained in the specified MAF file. Thus, to address the referee's point regarding GECKO validation we instead used the simTest collection of sequence files, downloaded from: http://compbio.soe.ucsc.edu/alignathon/data/simTest.seqs.tar.gz. We analysed the results of simMouse and simRat sequence comparisons using MUMmer and GECKO with the length parameter set to 20. We have used this parameter because after inspecting the MAF file we noticed that there was a significant number of short alignments.

In the comparison of both sequences from chr0, MUMmer obtained mostly short alignments with an average length of 24.84. In contrast, GECKO obtained much longer alignments with an average length of 95.76, and a maximum alignment length of 1831 versus a maximum of 107 reported by MUMmer. More info regarding alignment length distribution is provided in the image and table shown below.

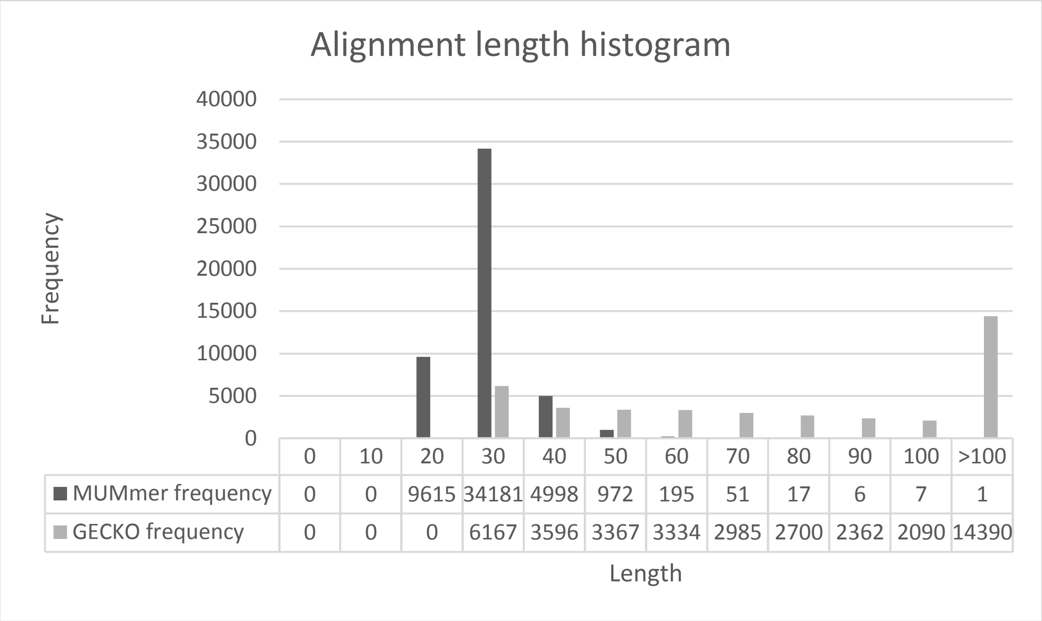

	0	10	20	30	40	50	60	70	80	90	100	>100
MUMmer frequency	0	0	9615	34181	4998	972	195	51	17	6	7	1
GECKO frequency	0	0	0	6167	3596	3367	3334	2985	2700	2362	2090	14390

	MUMmer	GECKO
max	107.00	1831.00
avg	24.84	95.76
std	5.80	76.67
min	20.00	21.00

Statistical values of the distribution of alignment lengths obtained in the comparison of chr0 using MUMmer and Gecko.

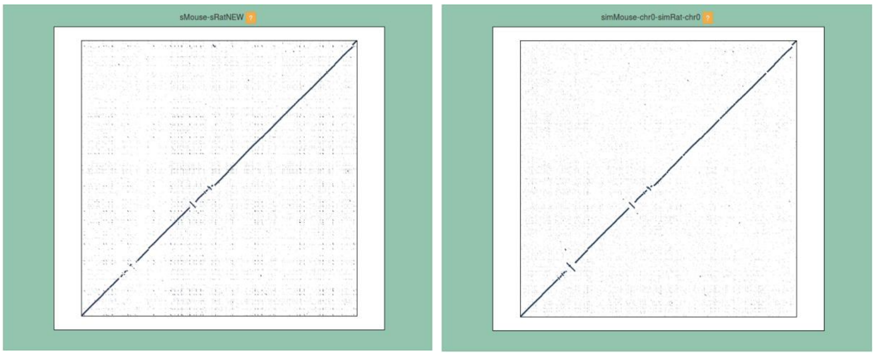

Dotplot representations of simMouse and simRat chr0 sequence comparisons using MUMmer (left) and Gecko (right).

The previous figure shows visual representations of the chr0 comparison of Mouse and Rat sequences with MUMmer and GECKO. In order to make it easier to compare the two methods, results were generated using the visualisation tool available at: http://chirimoyo.ac.uma.es/mrsymbiomathapp/pruebamg/.

The percentage of reverse strand fragments reported by GECKO was actually lower (16.61%) than that reported by MUMmer (28.76%). However, this is due to the higher number of noisy fragments generated by MUMmer as can be seen in Figure R1 (left); such fragments (in both forward and reverse strands) are longer in GECKO, in accordance with the longer alignments present in the MAF file.

The resulting GECKO file is available at: http://chirimoyo.ac.uma.es/gecko/files/simMouse-chr0-simRat-chr0.csv

The original output file of MUMmer was first transformed into our binary representation with the use of the mummer2frags program (compiled packages and instructions available from: http://chirimoyo.ac.uma.es/gecko/files/mummer2Frags.tar.gz) and then converted into a CSV file using the getInfo program included in the GECKO software suite (for more details about this function see reviewer comment 1.8). The final CSV file of MUMmer is available at: http://chirimoyo.ac.uma.es/gecko/files/sMouse-chr0-sRat-chr0-mummer.csv

A similar scenario was seen in the case of chr1:

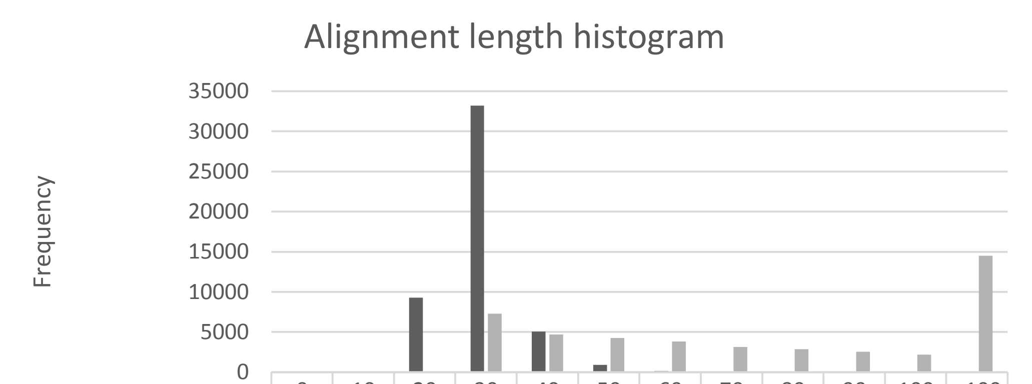

	0	10	20	30	40	50	60	70	80	90	100	>100
MUMmer frequency	0	0	9275	33186	5050	897	168	42	8	2	0	2
GECKO frequency	0	0	0	7298	4710	4276	3817	3153	2859	2552	2177	14506

Length

	MUMmer	GECKO
max	103.00	878.00
avg	24.83	90.53
Std	5.61	73.09
min	20.00	21.00

Statistical values of the distribution of alignment lengths obtained in the comparison of chr1 for mouse and rat.

Chr1 alignment dotplots were generated using the same visualisation tool as before. The two analyses can be downloaded from: http://chirimoyo.ac.uma.es/gecko/files/simMouse-chr1-simRat-chr1.csv (GECKO) and http://chirimoyo.ac.uma.es/gecko/files/sMouse-chr1-sRat-chr1-mummer.csv (MUMmer).

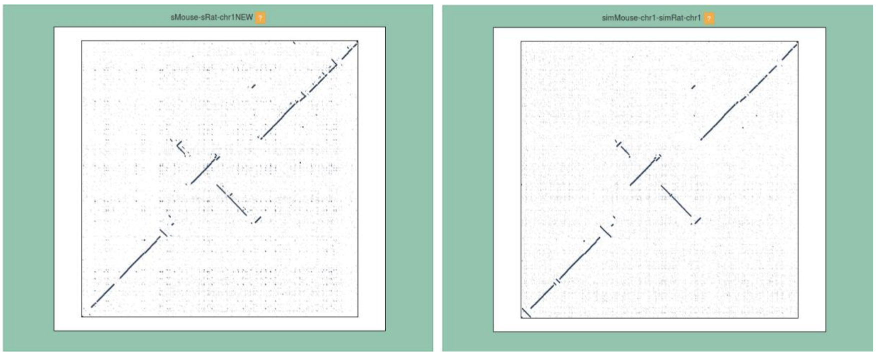

Dotplot representations of simMouse and simRat chr1 sequence alignments performed by MUMmer (left) and Gecko (right).

The execution time of the above exercises (chr0 and chr1 comparisons) was virtually identical (7.147 and 7.438 seconds per comparison in GECKO and MUMmer, respectively), however there were differences in their alignment length distribution. It is of interest to note the high frequency of small HSPs (especially in MUMmer) caused by using L=20 in both methods (and 16 as the K parameter value in GECKO). This would be expected to result in high coverage percentages albeit with high noise levels. In fact, coverage was 63.12% for GECKO and 15.48% for MUMmer in the chr0 sequence comparisons; and 63.17% for GECKO and 14.99% for MUMmer in the chr1 sequence comparisons. Differences in alignment length distribution were also noticed when calculating the coverage of the two programs when only considering HSPs longer than 30 residues. The coverage difference in this situation was even higher, with 61.92% coverage for GECKO and 3.47% for MUMmer with respect to chr0 (the difference can be seen graphically in Figure R3).

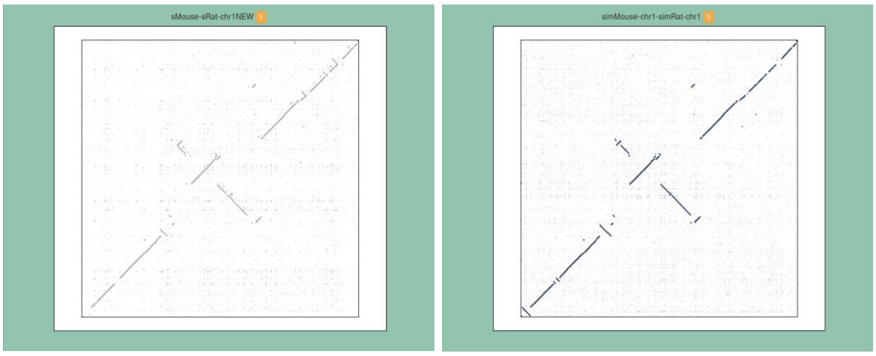

Dotplot representations of simMouse and simRat chr0 sequence alignments performed by MUMmer (left) and Gecko (right). Grey lines refer to filtered HSPs with less than 30 residues, while black lines refer to HSPs longer than 30. It is important to note the visibly higher number of grey filtered HSPs in the case of MUMmer.
